# Extended sequence context shapes mutational bias in *Escherichia coli*

**DOI:** 10.1073/pnas.2601345123

**Published:** 2026-06-03

**Authors:** Matthew J. Jago, Rowan Green, Maisie R. Czernuszka, Stepan Denisov, Rok Krašovec, Christopher G. Knight, Mato Lagator

**Affiliations:** ^a^ Division of Evolution, Infection and Genomic Sciences, School of Biological Sciences, Faculty of Biology, Medicine and Health, University of Manchester, Manchester M13 9NT, United Kingdom; ^b^ Department of Zoology, Faculty of Science, University of British Columbia, Vancouver, BC V6T 1Z4, Canada

**Keywords:** mutational bias, sequence context, mutational hotspots, DNA proofreading, mismatch repair

## Abstract

Understanding how sequence context influences the likelihood of mutation at a given genomic locus is critical for deciphering evolutionary processes. It is well established that the immediately adjacent bases influence mutation rates, but the role of more distal bases remains poorly understood. Here, we analyze over 100,000 mutations from 32 *Escherichia coli* mutation accumulation experiments, encompassing strains with varying DNA proofreading and mismatch repair capabilities. By quantifying the frequency of each nucleotide up to 6 bp around mutation sites, we reveal complex mutational biases that extend beyond the immediately adjacent bases and are unique to each type of base pair substitution. Furthermore, which sequence contexts contribute most to mutational bias depends on what repair mechanisms are active and which strand serves as the leading versus lagging template during replication. Mononucleotide runs are prominent mutational hotspots–we systematically characterize which types of runs are the most mutagenic, including a previously undescribed hotspot for G:C→C:G transversions that can increase their frequency by up to four orders of magnitude. Remarkably, extending our analysis to 1,000 bp from mutation sites reveals that sequence context can influence mutational bias over unexpectedly long distances. These findings expose the intricate interactions between extended sequence context and DNA repair systems that shape spontaneous mutagenesis, and shed light on the mechanistic origins and evolutionary consequences of mutational signatures.

## Significance

Knowing where in the genome mutations are most likely to occur is essential for predicting evolution. Mutation rates depend on interactions between DNA repair activity and the local DNA sequence context, but most studies only consider the two base pairs immediately flanking mutation sites. By analyzing over 100,000 mutations from wild-type and hypermutator *Escherichia coli*, we show that sequence context effects extend far beyond these immediate neighbors—in some cases influencing mutation rates hundreds of base pairs away. We also describe how different DNA repair systems modulate these effects, leaving distinct signatures in the mutational landscape. These findings reshape our understanding of where mutations occur and why, with implications for predicting bacterial evolution and describing the underlying mutational processes.

Base pair substitution (BPS) mutations are a fundamental source of genetic diversity that underpin evolution and facilitate adaptation (1). However, the relative frequency of each BPS (i.e., the mutational spectrum) varies dramatically between organisms (2) and depends on numerous factors (3). By determining which substitutions are more likely to occur, biases in the mutational spectrum influence evolutionary trajectories (4) and shape genomic features such as nucleotide (5) and codon (6) composition. Changes to the mutational spectrum can increase (7) or decrease (8) the likelihood of beneficial mutations and accelerate the acquisition of clinically important phenotypes, such as drug resistance in bacteria (9, 10) and cancer (11). Understanding the factors that bias mutational spectra is therefore central to predicting genome evolution and addressing key biomedical challenges.

Evaluating the mutational spectrum requires a large sample of mutations. These may be obtained experimentally by sequencing a defined locus after selecting for mutants with a detectable phenotypic change (3, 12, 13). This is particularly effective for identifying mutational hotspots in specific genes of interest, but the mutational spectrum varies between genes and is impacted by selection. Alternatively, mutation accumulation (MA) experiments involve whole-genome sequencing of multiple independent lineages propagated for thousands of generations (14–19). They capture genome-wide mutational spectra while largely mitigating the impact of selection on mutation identity. As such, MA experiments are well suited for evaluating how intrinsic mutational processes such as replication fidelity, DNA repair, and sequence context contribute to mutational bias.

MA experiments with *Escherichia coli* have also shown that environmental factors such as ultraviolet light (20), antibiotic treatment (16), or exogenous oxidative stress (21) can elevate the rate of specific mutation types. However, for BPS mutations that arise spontaneously, the genome-wide mutational spectrum is primarily determined by the intrinsic error profile of DNA polymerase (22), and further shaped by which DNA repair systems are present (14). For example, loss of genes involved in repairing oxidized nucleotides results in a bias toward the transversion mutations associated with this type of damage (15). In contrast, disrupting either the exonucleolytic proofreading ability of DNA polymerase (19) or the postreplicative mismatch repair (MMR) pathway (14, 18) dramatically elevates the rate of transition mutations. This is because DNA polymerase intrinsically produces transitions more often than transversions (23), while proofreading and MMR in turn correct transitions more efficiently (24). When both are functional, proofreading and MMR considerably reduce the skew toward transitions, and thereby shape biases in the mutational spectrum.

MA experiments have also demonstrated that the mutational spectrum is not uniform throughout the genome, as the frequency of BPS mutations differs between loci (14). A key determinant of site-specific mutation rates is the local sequence context: the identity of the two base pairs that immediately surround a mutating site. For instance, A:T→G:C transitions in *E. coli* occur twice as frequently when a C:G base pair lies directly 3’ of the A:T site (15). Sequence context effects are themselves shaped by DNA repair capability, e.g., the bias toward A:T→G:C transitions at sites with a 3’ C:G is significantly stronger in the absence of MMR (18). Thus, the local sequence context interacts with active DNA repair systems to modulate BPS rates across the genome. Strains with different DNA repair defects can therefore be identified by their specific mutational signature–the relative frequency of each BPS across different sequence contexts. This approach has been used to infer which mutational processes have shaped the evolution of bacterial lineages (25), and to determine the causes of tumorogenesis (26).

While the importance of the immediately adjacent base pairs to a given site’s mutational bias is well characterized, the influence of further nucleotides has received limited attention, and in bacteria has only been explored for a few specific motifs. For example, the sequences targeted by Dam and Dcm methylases (GATC and CCWGG respectively) are hotspots for mutations at the methylated base (14, 27). Furthermore, in several bacterial species, a T preceded by a run of three or more Gs is a hotspot for A:T→C:G transversions, with the nucleotides preceding the G tract also influencing the mutation rate (28, 29). However, a comprehensive analysis of the extended sequence context’s contribution to mutational bias in bacteria is lacking.

Here, we describe how nucleotides located two or more base pairs from a mutation site influence the rates of all BPS types, and how DNA repair systems both mask and generate context-dependent mutational biases. We collated data from 32 *E. coli* MA experiments (14–19), and grouped them according to the strains’ proofreading and MMR proficiency. By examining nucleotide frequencies up to 6 bp flanking each mutation site, we uncover complex sequence context effects that are dependent on the BPS type, repair background, and replication strand. We identify and characterize numerous mutagenic motifs, including a previously unrecognized hotspot for the rarest type of BPS, G:C→C:G transversions. Extending the analysis to 1,000 bp surrounding mutation sites shows that sequence context can contribute to mutational bias over much greater distances than generally appreciated. Together, these results refine our understanding of the mutational signatures associated with DNA proofreading and MMR while demonstrating the pervasive influence of extended sequence context on spontaneous mutagenesis in bacteria.

## Results

### Mutation Accumulation Data Used in This Study.

We analyzed data from 32 MA experiments (14–19) conducted under identical conditions across 19 different *E. coli* MG1655-derived strains (*SI Appendix*, Table S1), comprising 117,807 base pair substitutions (BPS). Strains were grouped according to their proficiency (+) or deficiency (−) for DNA proofreading and MMR (Table 1). Within each of the four repair groups, mutational spectra were highly consistent among strains (*SI Appendix*, Fig. S1). For each group, we calculated mutation rates for every BPS type: the two transitions (A:T→G:C, G:C→A:T) and the four transversions (A:T→C:G, G:C→T:A, A:T→T:A, G:C→C:G).

**Table 1.** Base pair substitution data from four DNA repair backgrounds

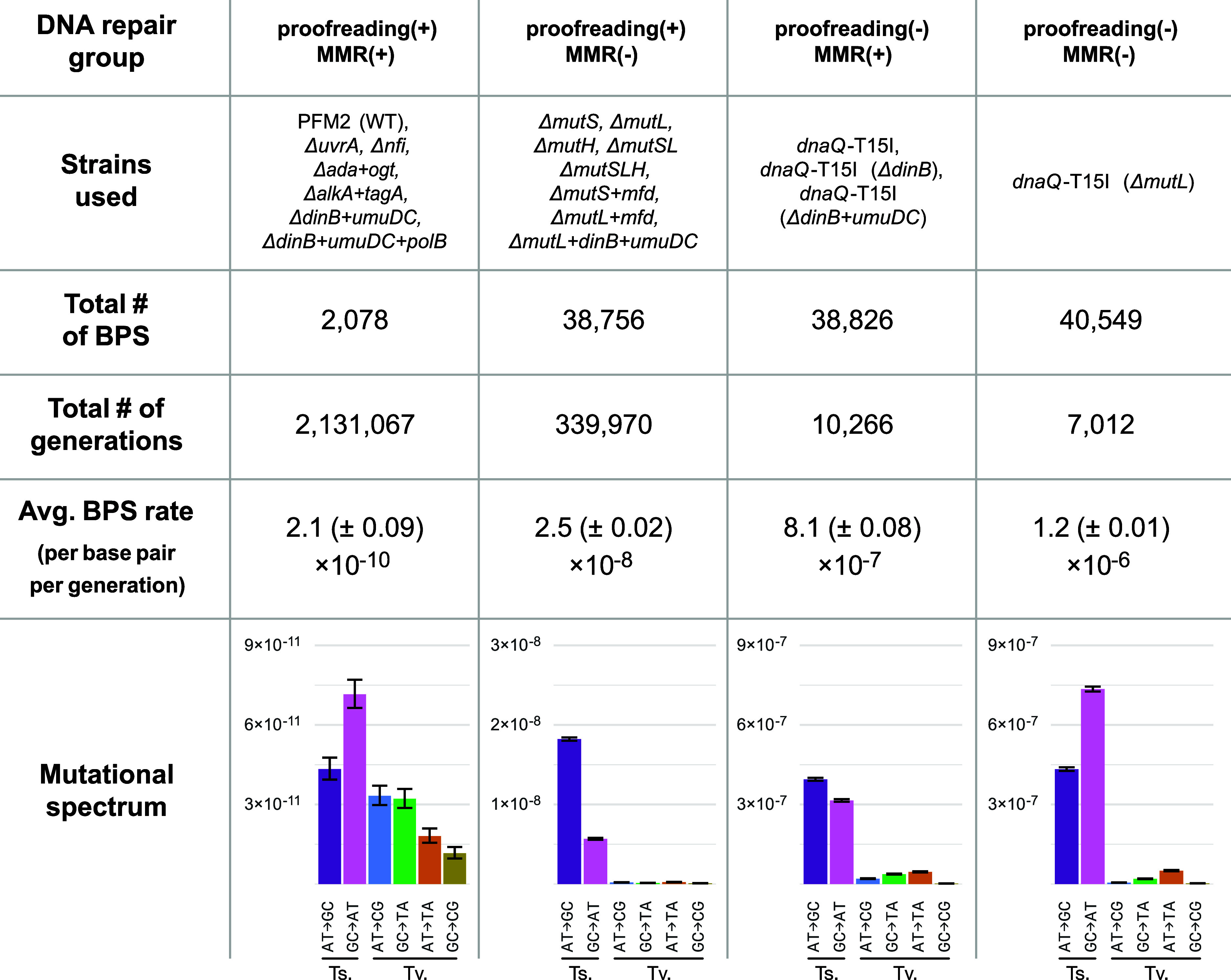

We collated BPS data from six published studies (14–19) comprising 32 mutation accumulation experiments across 19 *E. coli* strains, including the WT (PFM2) and various DNA repair gene knockouts (*SI Appendix*, Table S1). Strains were grouped according to proofreading and MMR proficiency (see *Methods* for details). For each group, the average BPS rate was calculated by pooling mutation counts across experiments then dividing by the total number of generations multiplied by the number of base pairs in the genome (4,639,675). Uncertainty was quantified using exact 95% Poisson CI on the pooled counts. The mutational spectrum shows the average rate for each type of BPS, the two transitions (Ts.) and the four transversions (Tv.), with error bars for the 95% CI.

The mutational spectrum varied considerably between the four repair groups (χ^2^_(15)_ = 22,294, *P* < 0.001) (Table 1). In proofreading(+) MMR(+) strains, the spectrum was moderately skewed toward G:C→A:T transitions (37%), while A:T→T:A (8%) and G:C→C:G (6%) transversions were the least common. Nonetheless, the spectrum remained relatively balanced compared to strains carrying DNA repair defects. Proofreading(+) MMR(−) strains exhibited a ~120-fold higher overall mutation rate and a spectrum dominated by transitions (97%), with a pronounced bias toward A:T→G:C transitions (~3.2× more common than G:C→A:T). Proofreading(−) MMR(+) strains displayed a ~4,000-fold higher mutation rate than the wildtype, transitions again dominating (87%) but with a less extreme bias toward A:T→G:C (~1.3× more common than G:C→A:T). The frequency of G:C→C:G transversions was extremely low (<0.5%) when either MMR or proofreading was knocked out, because polymerases infrequently generate this type of mismatch (30).

Finally, a proofreading(−) MMR(−) strain revealed the intrinsic mutational biases of DNA polymerase devoid of the primary repair pathways: nearly all mutations were transitions (94%), and within the transitions G:C→A:T occurred ~1.7× more often than A:T→G:C (Table 1). This ratio contrasts with strains lacking only one of proofreading *or* MMR, where A:T→G:C transitions predominate. As indicated by Niccum et al. (19), the transition rate at A:T versus G:C sites in the proofreading(−) MMR(−) strain closely resembles the ratio in proofreading(+) MMR(+) strains (χ^2^_(1)_ = 0.2, *P* = 0.65). This suggests an epistatic relationship between proofreading and MMR: individually, both systems preferentially repair A:T transitions, but when they are present together, G:C and A:T transitions are repaired with similar efficiency.

### Assigning 5’ versus 3’ Nucleotides.

Sequence context is typically evaluated by comparing how many mutations occurred at the central position of each possible trinucleotide (e.g., TAC). Reverse complementary trinucleotides (e.g., TAC and GTA) always appear together on opposite strands, and as such are treated as equivalent, because it is impossible to conclude from sequencing data which base templated the original mispair. For example, an A:T→G:C mutation could have come from an A:C mispair at a TAC site, or a T:G mispair at the GTA site on the opposite strand.

**Fig. 1.**
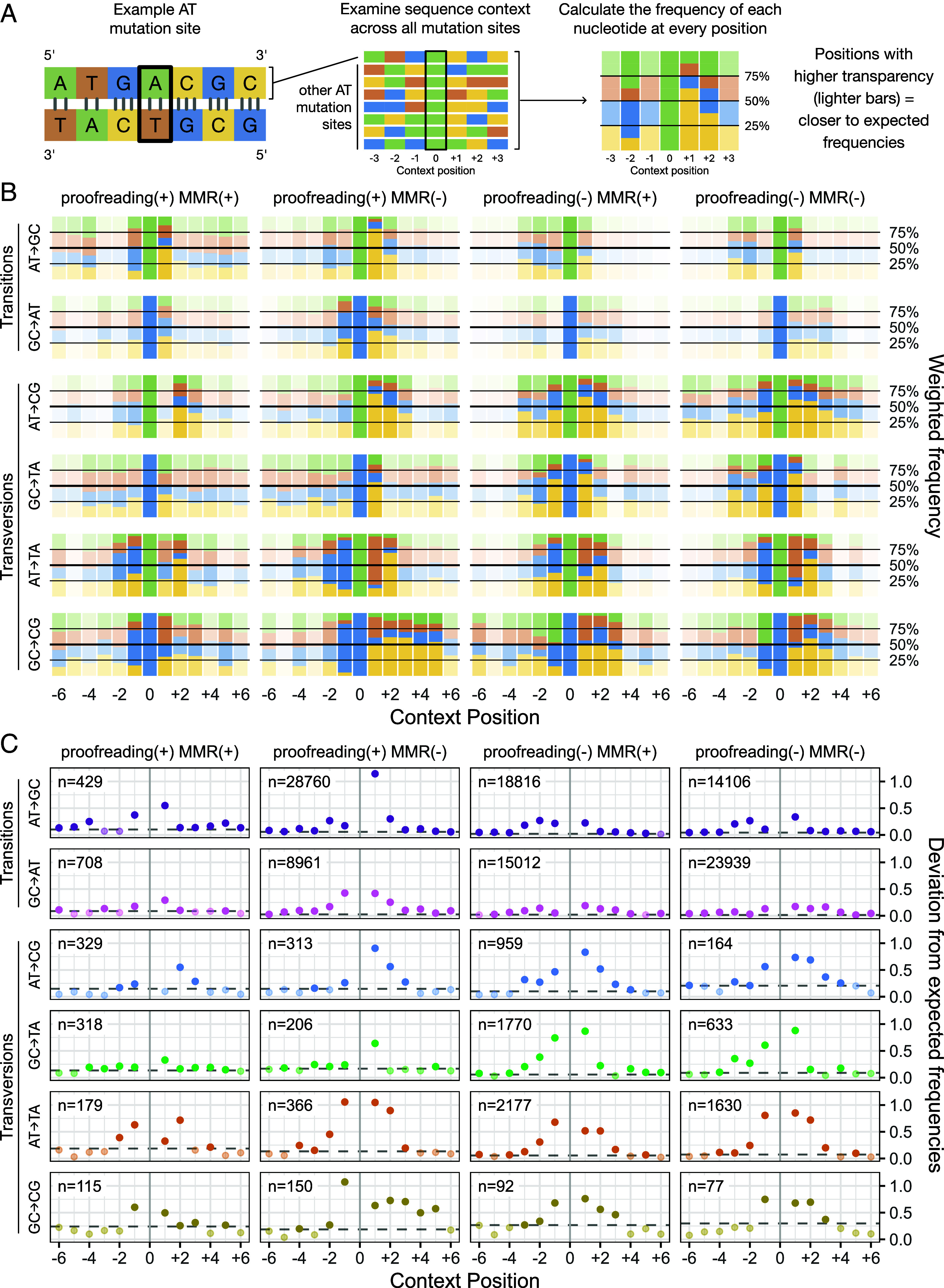
Sequence context biases up to ±6 base pairs away. (*A*) We examined the sequence context around every mutation site by calculating the frequency of A, T, G, and C at each context position within a ±6 bp window. (*B*) To visualize deviations from the overall genomic composition, we calculated expected nucleotide frequencies from the whole genome sequence (*SI Appendix*, Fig. S2) and weighted the observed frequencies such that 25% represents the null expectation for each nucleotide. Transparency at each position reflects the magnitude of deviation from expected frequencies, summed across all four nucleotides (darker = higher deviation). (*C*) The *y*-axis quantifies the sum deviation between observed and expected nucleotide frequencies. We also compared observed versus expected frequencies using chi-squared tests with false discovery rate correction. Positions where the deviation was significant (*P* < 0.05) are indicated with bold points (above the dashed line), while positions that were not significant are indicated with transparent points (below the dashed line). The number of mutations in our dataset (“n” in each plot) varies considerably between BPSs and repair backgrounds, so the significance threshold (dashed line) is not consistent between plots.

Consequently, whether the flanking nucleotides are designated as 5’ or 3’ relative to an A:T mutation site is arbitrarily decided by which base, A or T, is considered the focal nucleotide. Unless stated otherwise, we always consider the purine (A and G) as the mutated base pair’s focal nucleotide. Bases positioned 5’ of the purine are denoted with negative coordinates (−1, −2, …), while 3’ bases are denoted with positive coordinates (+1, +2, …) (Fig. 1*A*). This convention provides a consistent orientation for evaluating the directional influence of sequence context on mutation frequencies.

### The Extended Sequence Context (±6 bp) Influences Mutational Bias.

Although the local sequence context is recognized as a primary source of mutational bias in bacteria (31), motifs longer than three nucleotides have rarely been identified as significant (32). This is largely because the number of possible sequence combinations increases exponentially with motif length, making it difficult to detect significant differences in mutation rate between unique motifs.

To circumvent this issue, we examined the extended sequence context (±6 bp) around mutation sites without decomposing the data into distinct 13-bp motifs (~3.4 × 10^7^ possibilities). Instead, we considered each of the 12 context positions independently and calculated the relative frequency of A, T, G, and C at that position across all mutations (Fig. 1*B*). We then compared these observed nucleotide frequencies to those expected from the context surrounding every base in the genome (*SI Appendix*, Fig. S2), to quantify the degree of bias at each position (Fig. 1*C*). This approach captures how individual positions in the local sequence context influence the likelihood of a mutation at a given site. Although treating context positions independently meant we could not identify specific mutagenic motifs directly, it allowed us to evaluate context effects beyond the trinucleotide and highlighted which sequence features to investigate further.

The influence of extended sequence context varied markedly across different BPSs and repair backgrounds. While the immediately flanking bases (±1) often contributed most strongly to mutational bias, this was not always the case, and the extended context (±2 to 6) exerted a significant effect for all BPSs in every repair background (Fig. 1*C*). Extended context effects were generally stronger when one or both repair systems were inactive, indicating that proofreading and MMR can mask DNA polymerase’s intrinsic sequence biases. An exception was G:C→A:T transitions, for which context effects were weakest in proofreading(−) strains.

Overall, the extended context tended to influence transversions more than transitions, possibly reflecting their distinct mechanistic origins. Transversions are frequently caused by nucleotide damage, whereas transitions typically arise from polymerase misincorporation, tautomeric shifts, or spontaneous deamination (33). The pronounced variability in extended context biases across different BPS types and repair backgrounds demonstrates that mutational signatures persist beyond the trinucleotide, likely reflecting mutational mechanisms that depend on longer motifs.

### Known Mutational Hotspots Only Partially Explain Extended Context Bias.

Some of the extended context biases we observed (Fig. 1) can be attributed to mutational hotspots–specific sequence motifs with elevated mutation rates. Two well-known hotspots in *E. coli* are GATC and CCWGG, the target sequences for Dam and Dcm methylase, respectively (14, 27). Mutations arising from GATC methylation are most often A:T→T:A transversions because methylated adenine easily depurinates, and polymerase prefers to incorporate adenine opposite the abasic site (34). Conversely, Dcm activity typically leads to G:C→A:T transitions, as methylated cytosine readily deaminates into thymine (35). The GATC motif is clearly visible in Fig. 1*B* because it accounts for a substantial proportion of A:T→T:A transversions–as high as two-thirds in proofreading(+) MMR(−) strains (*SI Appendix*, Fig. S3). In contrast, CCWGG sites account for a much smaller fraction of G:C→A:T transitions, and consequently this motif is obscured in Fig. 1*B* by stronger context effects.

Recently, a T preceded by a run of three or more Gs was identified as a prominent hotspot for A:T→C:G transversions in DNA repair proficient bacteria (28, 29). The hotspot, which here appears as A followed by three or more Cs (i.e. AC_3+_) due to our purine-centric orientation, also persists in the absence of proofreading or MMR (Fig. 1*B*). In fact, the motif is least obvious for proofreading(+) MMR(+) strains, where C is overrepresented at the +2 and +3 positions but not +1. This is because, while AC_3+_ is a hotspot for A:T→C:G transversions, at AC_1_ sites there were significantly fewer mutations than expected in proofreading(+) MMR(+) strains (*SI Appendix*, Fig. S4*A*). At the +1 position, the AC_3+_ hotspot and AC_1_ coldspot effectively “cancel out,” such that the immediately 3’ nucleotide appears to not influence mutational bias (Fig. 1*C*)–a phenomenon that could only be detected by evaluating sequence context beyond the trinucleotide.

Interestingly, C nucleotides were also overrepresented in the 3’ context of G:C→C:G mutations in strains that lack proofreading or MMR (Fig. 1*B*). Evidently, this is because a 3’ mononucleotide run of Cs (i.e., GC_3+_) is also a prominent hotspot for G:C→C:G, accounting for 26 to 43% of these mutations in repair-deficient strains (*SI Appendix*, Fig. S4*B*). However, the hotspot was not identified in previous studies because it is negligible in proofreading(+) MMR(+) strains. Therefore, although proofreading and MMR are unable to prevent excess A:T→C:G mutations at AC_3+_ sites, both repair pathways together are highly effective at suppressing G:C→C:G mutations at GC_3+_ sites. We further examine mononucleotide run hotspots and their mechanisms in the following section.

The GATC, CCWGG, AC_3+_, and GC_3+_ hotspots substantially elevate local mutation rates and thereby shape mutational bias. However, the prevalence of mutations at these motifs may have obscured additional, subtler sequence contexts that also contribute to mutational bias. To uncover these, we recalculated context nucleotide frequencies after filtering out mutations at the mentioned hotspots (*SI Appendix*, Fig. S5). Context effects remained highly varied between different BPS and repair backgrounds, and several previously hidden mutagenic motifs emerged. Crucially, positions beyond the immediate ±1 nucleotides continued to exert significant influence on mutational bias. This demonstrates that other mutagenic processes, independent of methylase and mononucleotide run hotspots, have mutational signatures modulated by the extended sequence context.

### AC_3+_ and GC_3+_ Are the Strongest Mononucleotide Run Hotspots.

Mononucleotide runs are best known as hotspots for insertions or deletions rather than BPSs, because DNA polymerase is prone to slippage at repetitive sequences (36). Reannealing incorrectly generates a loop-out in either the nascent or template strand, resulting in an insertion or deletion, respectively. However, a BPS mutation can arise if strand alignment is restored by a second slippage event at the same run (Box 1). This phenomenon, known as transient misalignment, was demonstrated in vitro decades ago (37) but only recently confirmed in vivo using MA experiments in yeast (38). Transient misalignment likely contributes to the elevated BPS rate at mononucleotide runs in bacteria (18, 28, 29), but which BPS types and run motifs are most susceptible has not been systematically characterized.

Box 1. Transient misalignment can lead to BPS mutations

Mononucleotide runs are hotspots for insertion/deletion mutations because DNA polymerase can slip at repetitive sequences, causing the two strands to misalign and a base on either the template or nascent strand to loop-out. If the polymerase continues past the run, then the looped-out base leads to an insertion (nascent strand) or deletion (template strand). However, if the two strands realign before the polymerase has moved past the run, then a BPS mutation can be caused by this “transient misalignment.” Realignment causes the correctly paired nucleotide at the end of the run to be shunted down one position, which produces a mispair. If the looped-out base is on the nascent strand, then transient misalignment leads to a BPS at the adjacent base immediately downstream of the run, which mutates into the run nucleotide. If the looped-out base is on the template strand, then transient misalignment leads to a BPS at the final base in the run, where the run nucleotide mutates into the immediately adjacent nucleotide. In the diagram, bolding indicates where the mutation occurs. For more on the mutagenic mechanism of transient misalignment, see Lujan et al. (38).

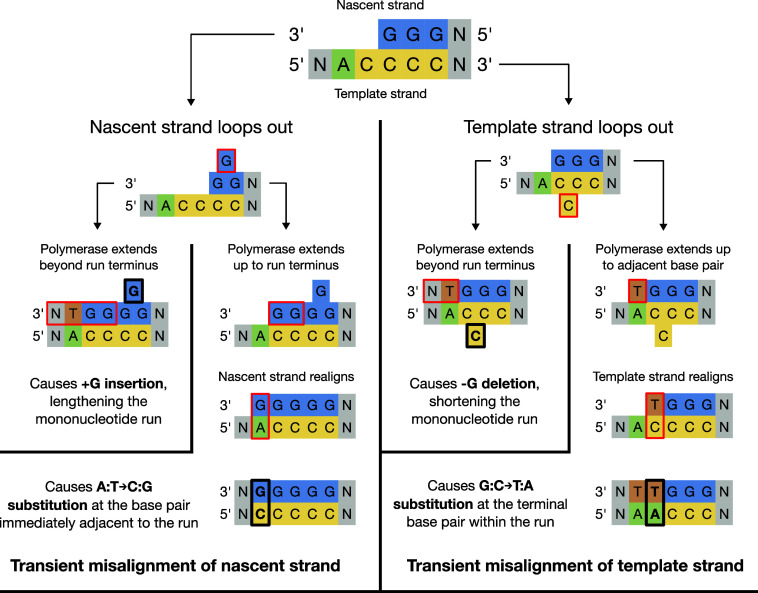

**Fig. 2.**
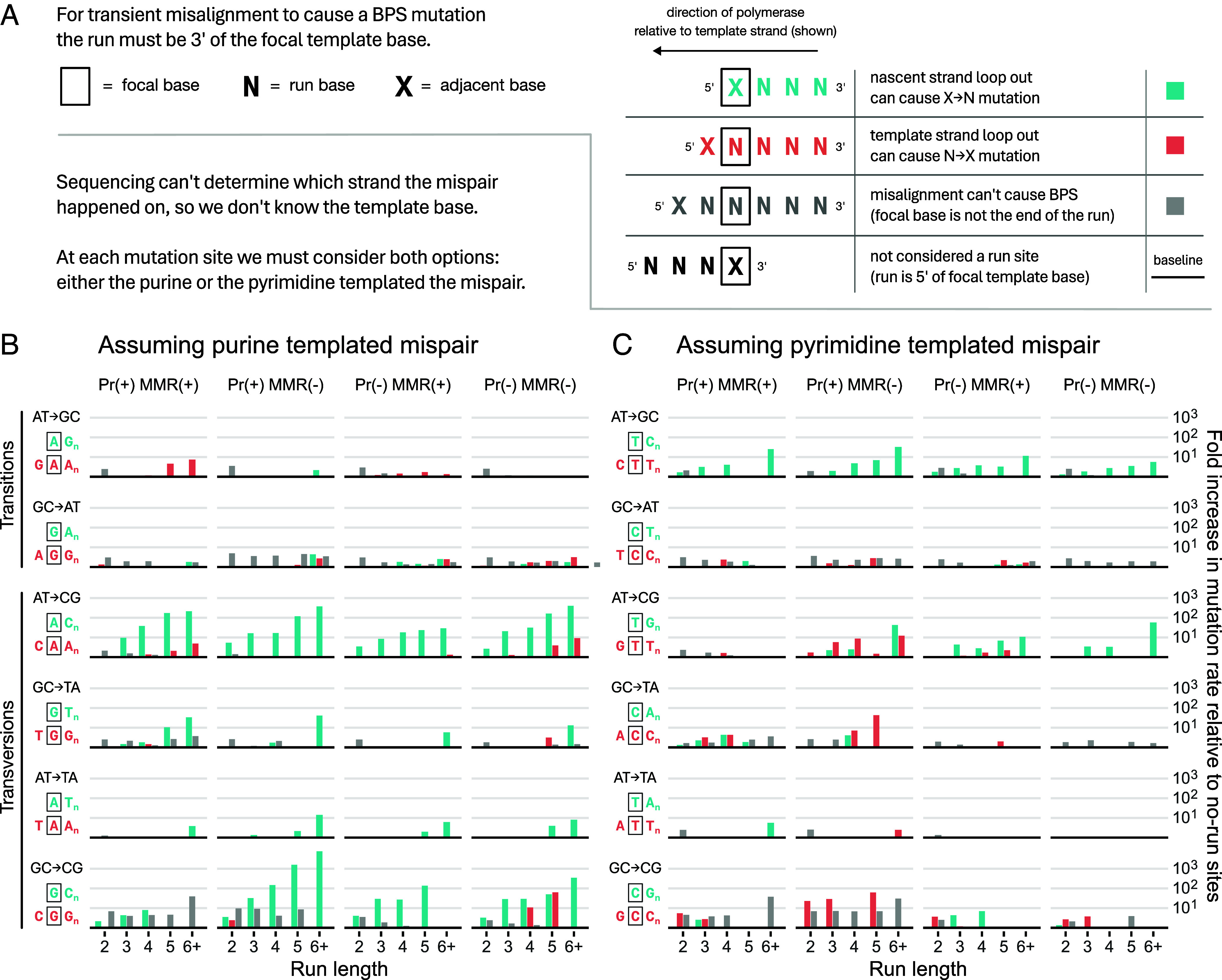
BPS hotspots that are consistent with transient misalignment. For every mutation site that was either within or adjacent to a ≥2 bp mononucleotide run, we determined if the BPS at that site was consistent with transient misalignment of either the nascent strand (blue) or template strand (red), or inconsistent with transient misalignment (gray). We then calculated the fold increase in mutation rate relative to sites that are not within or directly downstream of a run. (*A*) Whether a run is upstream or downstream of the mutation site depends on which strand the mispair happened on, something that whole genome sequencing cannot determine. We therefore performed this analysis twice, once assuming every mispair was templated by the strand with a purine at the focal site (*B*), and once assuming the pyrimidine templated (*C*). Therefore, a transient misalignment hotspot appearing in *B* suggests the mispairs at those sites were templated by the purine, whereas a hotspot appearing in *C* suggests the pyrimidine templated the mispairs. The mutation rates shown are relative to sites that are not within or immediately downstream of a ≥2 bp run; raw mutation rates for all sites are shown in *SI Appendix*, Fig. S6.

The AC_3+_ and GC_3+_ hotspots are both consistent with transient misalignment following a nascent strand loop-out, because the mutating base is immediately *adjacent* to the mononucleotide run. In contrast, a template strand loop-out would generate a BPS mutation at the terminal base *within* the run. To compare the prevalence of these mechanisms, we calculated the rate of each BPS at sites located within or adjacent to mononucleotide runs–distinguishing between sites that are consistent with transient misalignment of the nascent or template strand, versus sites that are incompatible with transient misalignment (Fig. 2).

Unsurprisingly, the strongest mononucleotide run hotspots were A:T→C:G transversions at AC_3+_ and G:C→C:G transversions at GC_3+_. For transient misalignment to cause a BPS mutation, the run must be upstream of the mutation site during replication, which for the template strand means the run is 3’ of the mutating nucleotide (Fig. 2*A*). Because both hotspots are characterized by a run of Cs 3’ of the purine, we can deduce that mispairs at AC_3+_ and GC_3+_ were templated by the purine (A and G) (Fig. 2*B*). The next strongest hotspot, for A:T→G:C transitions, was also consistent with a nascent strand loop-out, but with the pyrimidine templating the mispair (TC_3+_) (Fig. 2*C*). Notably, the three strongest mononucleotide run hotspots are all consistent with a run of C nucleotides on the template strand causing the nascent strand to loop-out.

AC_3+_ and GC_3+_ are the strongest hotspots because they drastically increase the rate of their respective BPS relative to sites without a run. Consequently, these hotspots account for a high proportion of A:T→C:G and G:C→C:G transversions at longer run lengths (*SI Appendix*, Fig. S6*A*). In contrast, a much smaller proportion of A:T→G:C transitions occurred at sites consistent with transient misalignment. However, because the overall frequency of A:T→G:C tended to be much higher than A:T→C:G or G:C→C:G, the actual mutation rate at TC_3+_ sites could be over 10-fold higher than AC_3+_ or GC_3+_ sites (*SI Appendix*, Fig. S6*B*). Therefore, realignment of a nascent strand loop-out at a TC_3+_ site is likely the most common transient misalignment event. This is supported by examining cases where the exact same mutation occurred at the same locus across several independent MA lineages (*SI Appendix*, Fig. S7). Almost all these hotspots were A:T→G:C transitions at TC_3+_ sites, although the mutation with the highest occurrence (nine lineages) was a G:C→C:G transversion at a GC_7_ site.

Overall, the majority of mononucleotide run hotspots were consistent with transient misalignment of the nascent strand, rather than the template strand (Fig. 2). The template strand hotspots we identified were weaker, and more dependent on longer run lengths or specific repair backgrounds. The strongest template strand hotspot was for G:C→C:G transversions assuming a pyrimidine templated the mispair (GCC_1+_) (Fig. 2*B*). Generally, nascent strand misalignment appears to be the dominant mechanism if a purine templates the mispair, while template strand misalignment prevails if a pyrimidine templates (*SI Appendix*, Fig. S6). However, this pattern is notably reversed for A:T→G:C transitions–the strong nascent strand hotspot (TC_3+_) depends on a pyrimidine template, and the much weaker template strand hotspot (GAA_4+_) depends on a purine template.

In some cases, we also found hotspots at run sites that are *not* compatible with transient misalignment (Fig. 2, grey bars), either because the site is not at the end of the run, or because transient misalignment would not cause the observed BPS mutation. Interestingly, the strongest hotspots in this category were also for G:C→C:G transversions. Given the rarity of G:C→C:G (Table 1), being within or adjacent to a run of G:C base pairs is therefore a key predictor of where this BPS will happen. Together, these results indicate that transient misalignment of the nascent strand is the strongest contributor to mutational bias at mononucleotide runs, capable of elevating the BPS rate over 1,000-fold. However, other mechanisms contribute to mutagenesis at runs, and which mechanisms predominate depends heavily on the BPS type, the repair background, and which nucleotide templates the mispair.

### G:C→C:G Mutational Bias Is Shaped by Interacting Mutagenic Sequence Contexts.

**Fig. 3.**
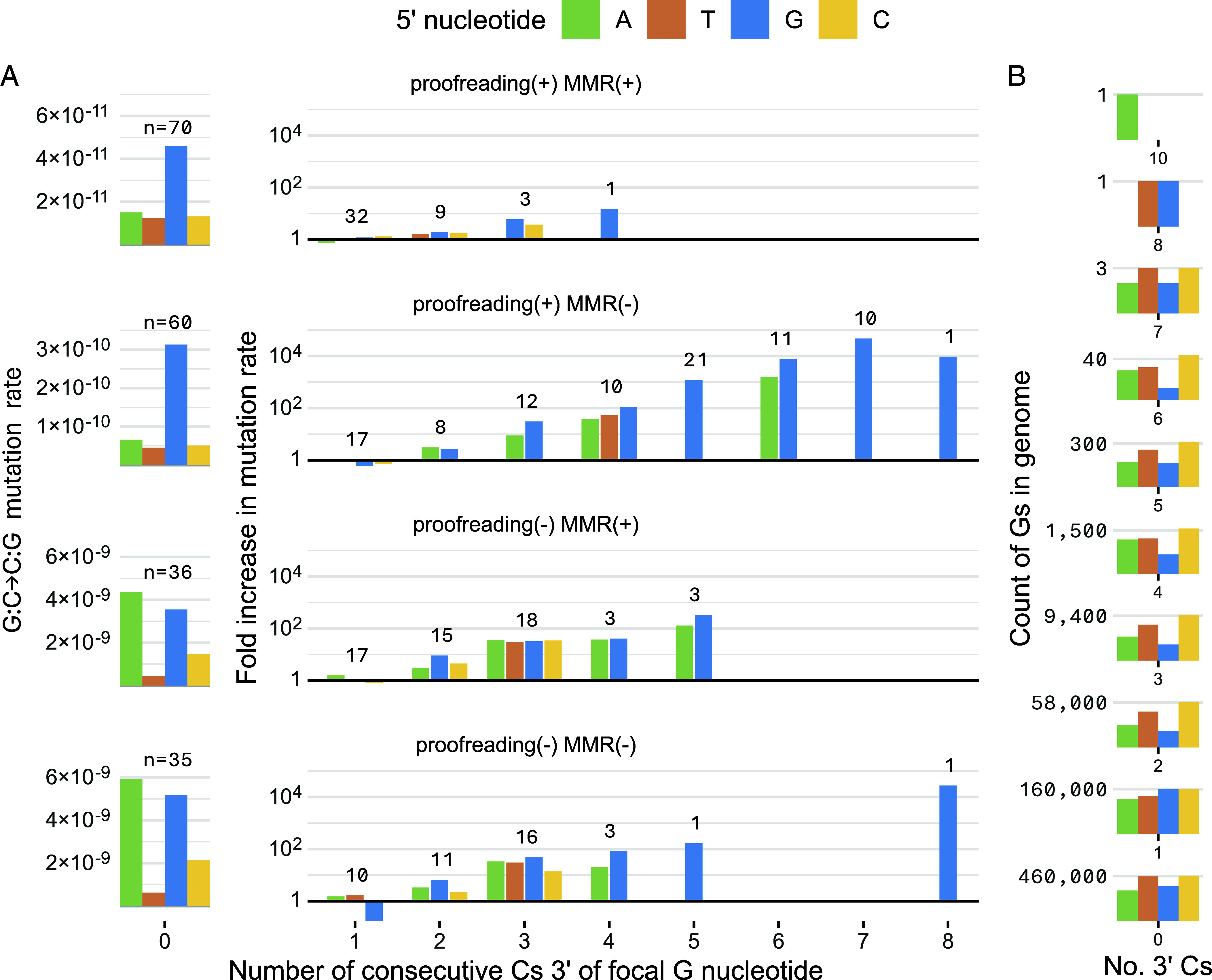
The GC_3+_ hotspot is influenced by the 5’ nucleotide. (*A*) The *Left*-most plots show the G:C→C:G mutation rate (per site per generation) at NGC_0_ sites, i.e., Gs without an immediately 3’ C. We calculated the rate separately depending on the identity of the 5’ nucleotide (color of the bar). The adjacent plots show the fold-increase in mutation rate at NGC_1+_ sites, relative to the corresponding NGC_0_ site. The *x*-axis shows the number of consecutive C nucleotides 3’ of the focal G. The numbers above indicate the total number of G:C→C:G mutations at GC_x_ sites. (*B*) The counts of all Gs in the genome split by the identity of the 5’ nucleotide (color) and the number of consecutive 3’ Cs (*x*-axis).

In addition to the GC_3+_ hotspot, another major contributor to G:C→C:G mutational bias is the identity of the immediately 5’ nucleotide (Fig. 1). To evaluate how this context bias interacts with the GC_3+_ hotspot, we calculated the G:C→C:G mutation rate at G sites stratified by the immediately 5’ nucleotide and the number of consecutive 3’ Cs. At sites without a 3’ C (i.e., GC_0_), the mutation rate was heavily influenced by the 5’ nucleotide (Fig. 3*A*). When proofreading was active, GGC_0_ sites had by far the highest mutation rate; in its absence, GGC_0_ and AGC_0_ both had elevated mutation rates.

Compared to GC_0_ sites, the mutation rate increased dramatically with longer runs of 3’ Cs–demonstrating the strength of the GC_3+_ hotspot (Fig. 3*A*). Beyond GC_2_, each additional C in the run increased the rate of G:C→C:G by up to 10-fold. Remarkably, the preference for a 5’ G seen with GC_0_ mutations persisted at sites with a 3’ run of Cs. Indeed, the 5’ nucleotide bias was even stronger for G:C→C:G mutations at longer runs: of the 48 mutations at sites with a run of five or more Cs, 46 were GGC_5+_ and 2 were AGC_5+_. In the most extreme case, proofreading(+) MMR(−) strains exhibited a ~50,000-fold higher rate for G:C→C:G transversions at GGC_7_ relative to GGC_0_, which in turn had a sixfold higher rate than TGC_0_. This interaction between the 5’ and 3’ nucleotide biases points to distinct mutagenic mechanisms that together create hotspots with even higher mutation rates.

GGC_3+_ and AGC_3+_ sites are also substantially less abundant in the genome than TGC_3+_ or CGC_3+_ sites, especially for longer run lengths (Fig. 3*B*). Possibly, having G or A immediately 5’ of a G nucleotide has been selected against because it elevates the G:C→C:G rate, or the higher rate simply means GC_3+_ sites mutate into CC_3+_ sites faster than the reverse. Regardless, the relative scarcity of GGC_3+_ and AGC_3+_ sites suggests that transient misalignment has influenced the genomic composition over generations by causing excess G:C→C:G transversions at these motifs. The observation that this hotspot is largely suppressed in proofreading(+) MMR(+) strains and strongest in MMR(−) strains is consistent with evidence that repeated cycles of losing and regaining MMR capability over *E. coli*’s evolutionary history have left an imprint on the genome (7, 39).

Furthering previous work in *Salmonella* (29), we also evaluated how the 5’ nucleotide influenced the strength of the AC_3+_ hotspot for A:T→C:G transversions (*SI Appendix*, Fig. S8). While the identity of the 5’ nucleotide did impact the AC_3+_ mutation rate, it was much less influential than at GC_3+_ sites, and which 5’ nucleotides were the most mutagenic was not the same between AC_0_ and AC_3+_ sites. These results indicate that, although the AC_3+_ and GC_3+_ hotspots are both consistent with transient misalignment of the nascent strand (Fig. 2), how this mutational mechanism interacts with the mechanism(s) responsible for 5’ biases depends on the BPS.

### DNA Strand Bias Can Interact with Sequence Context Bias.

**Fig. 4.**
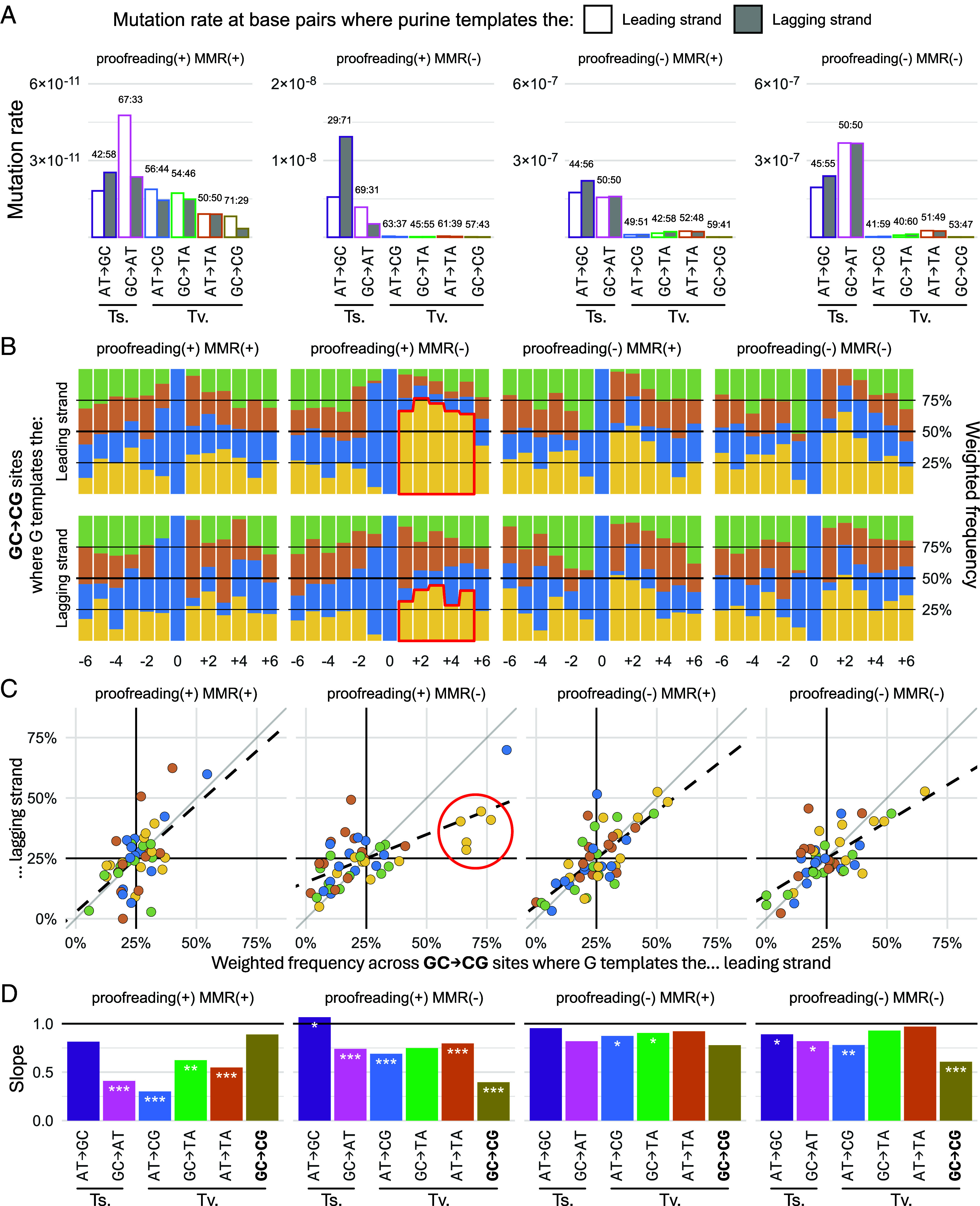
DNA strand influences extended sequence context biases. (*A*) The mutation rate (per base pair per generation) at a given site can depend on whether the purine resides on the leading strand or lagging strand during replication. (*B*) Sequence context nucleotide frequencies (as in Fig. 1*B*) for G:C→C:G mutations, split between sites where the purine (G) templates the leading versus the lagging strand. Color indicates the nucleotide: green = A, orange = T, blue = G, yellow = C. The bars outlined with red in proofreading(+) MMR(−) indicate the overrepresentation of 3’ C nucleotides caused by the GC_3+_ hotspot. These bars map to the points enclosed by a red circle in *C*) Direct comparison of the sequence context nucleotide frequencies at G:C→C:G sites where G templates the leading versus lagging strand. Each point represents the weighted frequency of one nucleotide (A, T, G, or C) at one position (−6 to +6) across all mutation sites. The dashed line represents the line of best fit from linear regression. If the nucleotide frequencies were identical between sites where the purine templates the leading versus lagging strand, then the line of best fit would have a slope of 1 and an R^2^ value of 1. Shown are only the plots for G:C→C:G mutations, *SI Appendix*, Fig. S9 shows the plots for every BPS and repair group. (*D*) The slope of the line of best fit for every BPS and repair group (taken from *C* and *SI Appendix*, Fig. S9). The slope reflects the relative strength of sequence context effects between strands: values <1 indicate that context contributes more to mutational bias at sites where the purine templates the leading strand, values >1 indicate the opposite. Stars indicate the results of linear hypothesis tests to determine if the slope is significantly different from 1 after false discovery rate correction (***P < 0.001, **P < 0.01, *P < 0.05). *SI Appendix*, Table S3 shows all slope and R^2^ values, along with the slope *P* values from the linear models.

During DNA replication, the two template strands are copied using different mechanisms. On the “leading” strand template (LDST), DNA polymerase binds once at the replication origin and synthesizes continuously. In contrast, the “lagging” strand template (LGST) is replicated discontinuously through repeated cycles of polymerase binding and dissociating. Replication of the LGST is thought to exhibit higher fidelity than the LDST because the frequent dissociation of polymerase facilitates proofreading (40, 41). The discrepancy in fidelity between strands also introduces mutational bias: the BPS rate at a given site can depend on which base (purine or pyrimidine) resides on the LDST versus LGST (14, 18, 31). These strand-dependent mutational biases varied markedly between BPS types and strains with different repair capabilities (Fig. 4*A*). Strand biases were generally weaker when proofreading was disabled–consistent with proofreading following polymerase dissociation being responsible for the difference in replication fidelity between strands (41).

While strand-dependent mutation rates are well established (14, 18, 31), whether the extended sequence context affects mutational bias in a strand-dependent manner has not been comprehensively explored. To test this, we compared nucleotide frequencies at the ±6 bp context positions between sites where the purine was on the LDST versus LGST (Fig. 4 *B*–*D* and *SI Appendix*, Fig. S9). We found that the influence of strand on sequence context biases depended on the type of BPS and the repair background. In some cases, sequence context biases were similar regardless of strand orientation, in others, the strength and/or identity of context biases varied between sites with the purine on the LDST versus LGST. For example, with G:C→C:G transversions in proofreading(+) MMR(−) strains, the preference for having Cs 3’ of the G was much stronger when G resides on the LDST (Fig. 4 *B* and *C*). This is because the mutation rate at the GC_3+_ hotspot (see previous section) was higher during leading strand replication (*SI Appendix*, Fig. S10). The same trend was reported for the AC_3+_ hotspot, which is stronger at sites with the purine (A) on the LDST (28). For both hotspots, the influence of strand was much weaker in proofreading(−) strains (*SI Appendix*, Fig. S10), consistent with elevated proofreading activity during LGST replication being responsible for the strand asymmetry.

Strand-dependent effects were often more pronounced in proofreading(+) strains, and when strand had a significant influence, context biases were nearly always stronger at sites where the purine resides on the LDST (Fig. 4*D*). Interestingly, strand asymmetry in context biases generally did not mirror the overall strand bias in mutation rates (Fig. 4*A*). For example, in proofreading(+) MMR(−) strains, the A:T→G:C mutation rate was considerably higher when A was on the LDST, but strand had a relatively minimal influence on the context biases. This decoupling suggests that the mechanisms driving strand-specific mutation rates and those generating strand-specific context effects are at least partly distinct. In other words, while strand bias in mutation rates (Fig. 4*A*) likely reflects differences in proofreading-efficiency, strand bias in sequence context effects (Fig. 4*D*) also depends on other factors. These findings highlight that “strand bias” is not a single phenomenon but a composite outcome of replication dynamics, repair mechanisms, and the extended sequence context.

### Sequence Context up to 1,000 Base Pairs Away Contributes to Mutational Bias.

**Fig. 5.**
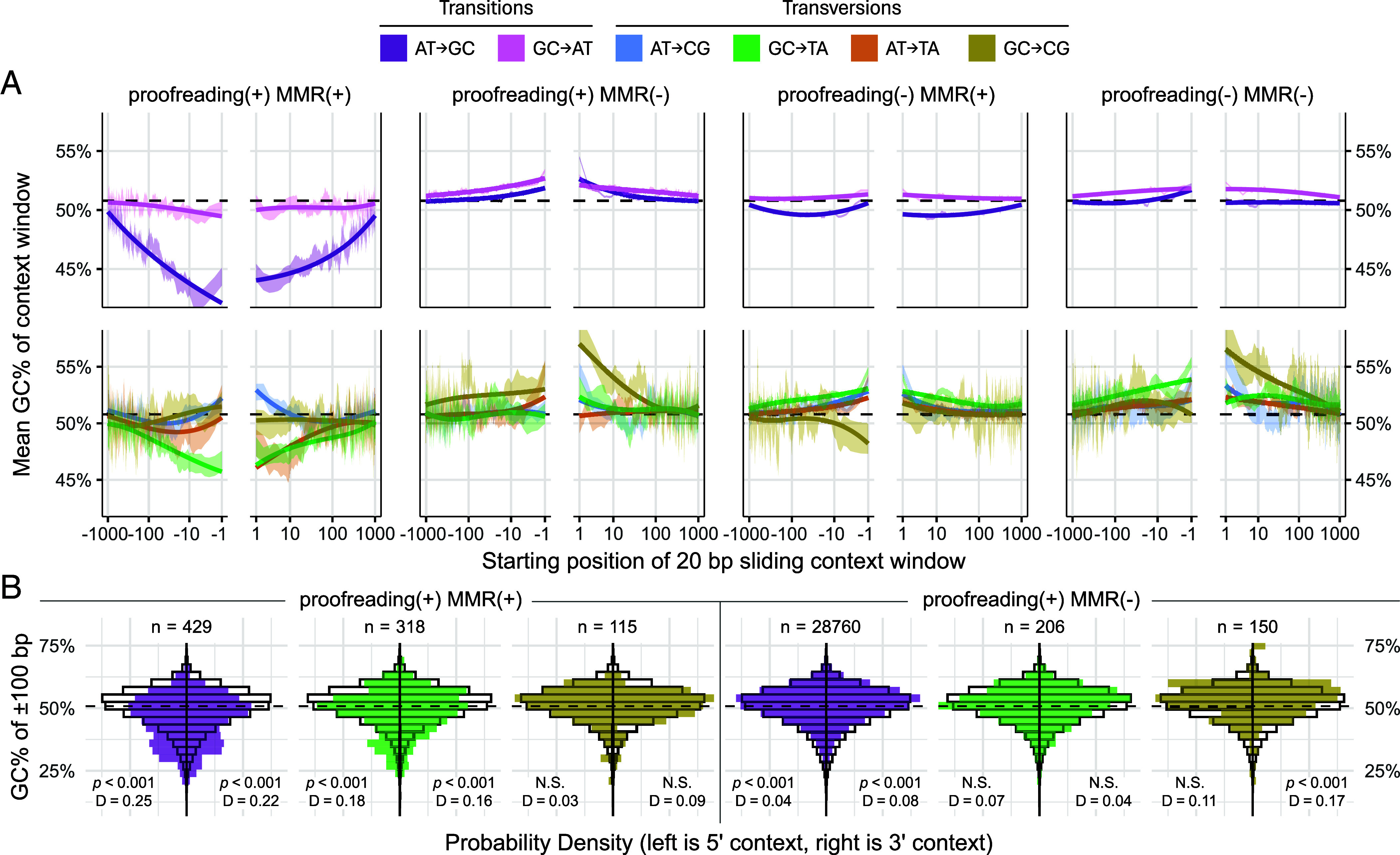
GC-content up to 1,000 bp away contributes to mutational bias. (*A*) The y-axis shows the mean GC% across all mutation sites of a 20 bp sliding context window that starts from ±1 to ±20 and ends with ±981 to ±1,000. The line shows a LOESS-smoothed fit of the mean GC% values and the shaded ribbon indicates the SEM for each context window. The horizontal dashed line shows the overall GC% of the *E.* coli genome (0.508). Transitions (*Top*) and transversions (*Bottom*) are shown separately for visual clarity. (*B*) Histograms of the probability density function for GC% values across all mutations for the −1 to −100 bp (*Left*) and +1 to +100 bp (*Right*) context windows. The black and white outline represents the null expectation from the same context windows around every site in the genome. We performed Kolmogorov–Smirnov tests with false discovery rate correction to compare the distributions to the null expectation (adjusted *P-*value and test statistic shown). This figure shows A:T→G:C transitions, A:T→C:G transversions, and G:C→C:G transversions for proofreading(+) MMR(+) and proofreading(+) MMR(−) strains, all BPSs and repair backgrounds are shown in *SI Appendix*, Fig. S12.

Given the importance of nucleotides up to ±6 bp away from mutation sites, we next asked whether context effects persist over even greater distances. In eukaryotes, the GC-content (GC%) of the wider sequence context can influence mutagenesis over kilobase scales (42, 43). To evaluate whether similar biases exist in *E. coli*, we calculated the average GC% of a 20 bp sliding context window, extending up to 1,000 bp away from each mutation site in either the 5’ or 3’ direction (Fig. 5*A*). If only the nearby nucleotides contribute to mutational bias, the average GC% surrounding mutation sites should rapidly converge to the genome-wide value (50.8%). In most cases this expectation held–however, several BPS types exhibited significant GC% biases that persisted hundreds of bp away from the mutation sites.

In proofreading(+) MMR(+) strains, the sequence context around A:T→G:C transitions and G:C→T:A transversions on average had lower GC% than expected (Fig. 5*A*). While the severity diminished with distance, for A:T→G:C transitions this bias extended up to ±1,000 bp from mutation sites, indicating that broader regional composition can influence mutational bias in bacteria. Strikingly, the bias toward AT-rich regions disappeared entirely in MMR(−) strains, suggesting that it arose because MMR preferentially repairs mismatches in GC-rich regions. This could also explain why the bias is strongest for A:T→G:C transitions, which are repaired by MMR at a much higher rate than any other BPS (Table 1).

In MMR(−) strains, the BPS most influenced by GC% was G:C→C:G transversions, which were more frequent in GC-rich regions (Fig. 5*A*). Interestingly, the strength of the bias and the distance it extended from mutation sites varied between the 5’ and 3’ directions, demonstrating that directionality can also influence distant context effects. Surprisingly, the mutational bias for higher GC% was not caused by the GC_3+_ hotspot, as it was also observed with G:C→C:G mutations that did not have a 3’ run of Cs (*SI Appendix*, Fig. S11). Because this bias appeared only when MMR was inactive–regardless of proofreading status–it likely reflects intrinsic polymerase behavior that is masked by MMR.

To directly evaluate which genomic regions were enriched for specific BPS types, we compared the distribution of GC% values from the ±1 to 100 bp context regions around each mutation site to the GC% distribution of the same contexts around every site in the genome (Fig. 5*B* and *SI Appendix*, Fig. S12). We found that the bias toward mutations within GC-poor contexts in proofreading(+) MMR(+) strains comes from excess mutations in regions with very low GC% (~25 to 40%). In contrast, the G:C→C:G bias in MMR-deficient strains is due to excess mutations in regions with slightly elevated GC% (~51 to 60%). Notably, the influence of GC% on mutagenesis was highly varied between different BPS and repair backgrounds, indicating complex interactions between GC% and other sources of mutational bias.

## Discussion

By analyzing over 100,000 BPS mutations in *E. coli*, we revealed that sequence context well beyond the immediately adjacent bases exerts a major influence on mutational bias, and that these effects vary sharply depending on both the type of BPS and which DNA repair mechanisms are active. We showed this by characterizing the mutational signatures of both proofreading and MMR at the individual context positions up to ±6 bp around mutation sites (Fig. 1). Trinucleotide (i.e., ±1) context signatures have recently been used to discern which mutational processes shape evolution in *E. coli* (25), and to determine which strains of pathogenic *Pseudomonas aeruginosa* are most likely to acquire multi-drug resistance (10). Our results demonstrate that accounting for the extended sequence context results in more precise mutational signatures, further improving the ability to describe and predict bacterial evolution, as human and viral studies have suggested (44–46).

We identified multiple mutagenic motifs, including a mononucleotide run hotspot (GC_3+_) that increased G:C→C:G transversions by up to four orders of magnitude in the absence of MMR (Figs. 2*B* and 3*B*). Another run hotspot (TC_3+_) elevated A:T→G:C transitions even when both MMR and proofreading were active (Fig. 2*C* and *SI Appendix*, Fig. S6). BPS mutations at mononucleotide runs have long been proposed to arise through transient misalignment (18, 37, 47), but only recently have studies begun to characterize its mutational signatures in detail (28, 29, 38). Our analysis strongly supports transient misalignment as a contributor to mutational bias, but only for certain BPS types and depending on the DNA strand. Which BPS is caused by transient misalignment is determined by the sequence context, implying the event is more frequent at some run motifs than others. We found that the strongest trigger for transient misalignment was a run of C nucleotides on the template strand, which causes the nascent strand to loop-out (Fig. 2).

MA experiments are well suited for identifying hotspots like mononucleotide runs that recur throughout the genome. In contrast, the large number of potential mutation sites and relatively few replicates mean MA is unlikely to detect rare hotspots that only exist at a single genomic locus. Such hotspots, which can heavily influence evolutionary outcomes, are often identified through reporter gene assays (48, 49). The smaller target space of these assays helps find more complex hotspots that depend on the extended sequence context forming secondary DNA structures, such as quasipalindrome sequences (50). Across the entire collated dataset, we nevertheless identified several genomic loci that mutated repeatedly (*SI Appendix*, Fig. S7), most of which are consistent with transient misalignment–further indicating a prominent role in spontaneous mutagenesis.

We also explored how DNA strand orientation (whether the purine or the pyrimidine templates leading versus lagging strand replication at a given position) not only influences mutation rate but also affects the identity and strength of mutagenic sequence contexts (Fig. 4). Notably, context effects were nearly always stronger at sites where the purine templates the leading strand–a phenomenon especially evident for G:C→C:G transversions, largely because of the GC_3+_ hotspot. Assuming mutations at the hotspot were caused by transient misalignment, then the purine (G) would have templated the mispair. This suggests the hotspot is stronger during leading strand replication (*SI Appendix*, Fig. S10), which is supportive of previous evidence that fidelity is higher during lagging strand synthesis (40). Additionally, the diminished strand bias in proofreading(−) strains is consistent with the hypothesis that lagging strand synthesis has higher fidelity because the increased rate of polymerase dissociation provides more opportunity for proofreading (41).

The various strand and context biases of G:C→C:G transversions are a recurring theme in this work, even though they were the least frequent type of BPS in all repair backgrounds. Indeed, their rarity may partly be explained by their exceptionally strong and specific context biases. G:C→C:G mutations are not only more frequent in specific sequence contexts, but also under certain cellular conditions. For example, constitutive expression of the SOS response elevates G:C→C:G mutation rates ~160-fold, a higher increase than any other BPS (32). Cellular state can also influence sequence context effects: G:C→C:G exhibited the strongest trinucleotide context biases in the constitutive SOS strains (32), even though the GC_3+_ hotspot remained fully suppressed by proofreading and MMR (*SI Appendix*, Fig. S13). Thus, the highly specific relationship between repair capability, cellular conditions, and sequence context that gives rise to elevated G:C→C:G rates suggests that the positioning of this BPS can serve as a characteristic signature of mutational phenotypes, despite its rarity.

Finally, our observation that the frequency of certain BPS types depends on the GC-content of the wider ±1,000 bp region (Fig. 5) points to long-range mechanistic biases in DNA replication and repair that have not been reported previously for bacteria. Most prominently, MMR appears less able to correct mispairs in AT-rich regions, particularly those leading to A:T→G:C transitions. However, *E. coli* ChIP-seq experiments for the MMR protein MutL bound to mispairs found it was enriched in regions with higher AT-content (51). This may suggest that excess mutations in AT-rich regions are not due to reduced recognition of the mispairs by MMR, but rather due to reduced efficiency of repair once recognized. Therefore, the enrichment of MutL proteins in AT-rich regions could have evolved to partially compensate for lower repair efficiency.

By collating the largest available BPS dataset from *E. coli* MA studies and employing unique analytical approaches, we have characterized how sequence context contributes to mutational bias with greater range and resolution than achieved previously. Our results show that every type of BPS is influenced by the extended sequence context, and in some cases even the context hundreds of base pairs away can affect mutation rates. We also demonstrate how proofreading and mismatch repair systems leave distinct mutational signatures by modulating these effects–providing insight into several mutational processes that drive genetic change through their complex interactions. Describing how the extended sequence context influences the identity and location of base pair substitutions furthers our understanding of spontaneous mutagenesis and brings us closer to predicting evolutionary outcomes.

## Methods

For more detail, please see *SI Appendix, Supplementary Methods*.

### Collating Mutation Accumulation Data.

We compiled BPS data from 32 mutation accumulation (MA) experiments across six published studies (14–19). All experiments were performed using Luria-Bertani agar and the PFM2 strain of *Escherichia coli*, a prototrophic derivative of K-12 MG1655 that carries a functional copy of *rpoS*, as described in Lee et al. (14). Each experiment included ~20 to 80 independent lineages that were passaged every day by streaking onto a fresh agar plate and randomly selecting a single colony. At the end of an experiment, one colony from each lineage was isolated for whole genome sequencing to identify mutations. The daily single-cell bottleneck means the effective population size for each lineage was one, maximizing genetic drift and largely mitigating the influence of selection on which mutations achieved fixation. While selection acting during colony growth can still introduce a degree of bias (52), the ratio of synonymous to nonsynonymous mutations in these studies suggests that selection was minimal (14, 15).

The entire collated BPS dataset is available in Dataset S1. As in the original studies, the genomic position of each mutation maps to the *E. coli* K-12 MG1655 reference sequence (NC_000913.3).

### Grouping Strains According to Proofreading and MMR Proficiency.

While all strains used in this study were derived from *E. coli* PFM2, most carried deletions of one or more DNA repair genes (Table 1 and *SI Appendix*, Table S1). Strains that lacked one or more of the key mismatch repair genes (*mutS*, *mutL*, and *mutH*) were classified as MMR(−), while proofreading(−) strains carried the *mutD5* allele of *dnaQ*, a T15I substitution that reduces proofreading activity for the primary replicative polymerase (DNA pol III) by 98%. Some strains lacked other DNA repair genes such as *uvrA, nfi, ada, ogt, alkA, tagA, or mfd*, and some lacked other polymerases like DNA pol II (*polB*), IV (*dinB*), or V (*umuDC*). However, these genes primarily operate under stressful conditions or in response to exogenous mutagens, so their absence has minimal influence on the mutational spectrum in MA experiments (15, 18, 19). Accordingly, we combined data from different strains based solely on their capacity for DNA proofreading and mismatch repair, which are the main pathways responsible for preventing BPS mutations (24).

This grouping strategy is consistent with previous analyses of trinucleotide sequence context (18, 25), and mutational spectra were highly consistent between strains within the same repair group (*SI Appendix*, Fig. S1). Furthermore, chi-squared tests on BPS counts across independent experiments show that the variation between different strains within the same repair group was comparable to the variation between different experiments performed with the same strain (*SI Appendix*, Table S2), supporting the decision to aggregate data based on proofreading and MMR proficiency. However, this grouping may have obscured some sequence context effects that are dependent on repair pathways other than proofreading or MMR.

### Evaluating Sequence Context Nucleotide Frequencies.

To calculate nucleotide frequencies, we divided the number of times a nucleotide was present at a given context position by the total number of mutations–separately for all six BPSs in the four DNA repair backgrounds (Fig. 1). To prevent the results from being skewed by the sequence of the genome itself, we weighted the observed nucleotide frequencies from mutation sites by the expected nucleotide frequencies from the context around every site in the genome (*SI Appendix*, Fig. S2). We calculated how much the observed frequencies deviated from the expected as $\sum_{n\in \{A,T,G,C\}}\left|f_{\mathrm{obs}}\left(n\right)-f_{\exp}\left(n\right)\right|$, where $f_{\mathrm{obs}}$ is the observed frequency and $f_{\exp}$ is the expected frequency. The sum deviation value for each context position is represented by transparency in Fig. 1*B* and the y-axis in Fig. 1*C*–the theoretical maximum is 1.5, corresponding to one nucleotide at 100% frequency and the other three at 0% frequency. Finally, we also performed chi-squared tests with false discovery rate correction to determine if the nucleotide frequencies at a given context position were significantly different (*P* < 0.05) from the genomic expectation. The results of these tests are summarized in Fig. 1*C* and Dataset S2 includes the test statistic, raw, and corrected *P* values for every test.

## Supplementary Material

Appendix 01 (PDF)

Dataset S01 (XLSX)

Dataset S02 (XLSX)

## Data Availability

The full dataset of BPS mutations collated for this study is available in Dataset S1. The scripts used to analyze the BPS data are available on GitHub (https://github.com/Lagator-Group/extended-sequence-context) (53).
