# Supplementary material for: Extended sequence context shapes mutational bias in Escherichia coli
Source: Proc Natl Acad Sci U S A. 2026 Jun 3;123(23):e2601345123. doi: 10.1073/pnas.2601345123 (PMC13250602; doi:10.1073/pnas.2601345123)
Supplement: Supplementary file 1 — Appendix 01 (PDF) [file pnas.2601345123.sapp.pdf]

Supporting Information for:
Extended sequence context shapes mutational bias in *Escherichia coli*

SUPPLEMENTARY METHODS **2**

Collating mutation accumulation data 2

Grouping strains according to proofreading and MMR proficiency 2

Evaluating sequence context nucleotide frequencies 3

Comparing leading versus lagging strand template 4

Calculating mutation rates at mononucleotide runs 5

Determining how regional GC-content influences mutagenesis 6

Programs and packages 6

SUPPLEMENTARY FIGURES **7**

Figure S1. Mutational spectrum of the strains comprising each DNA repair group 7

Figure S2. Average genomic sequence context 8

Figure S3. Rate and proportion of mutations at GATC and CCWGG hotspots 9

Figure S4. Proportion of mutations at AC_n and GC_n sites 10

Figure S5. Nucleotide frequencies after excluding the strongest mutational hotspots 11

Figure S6. Proportions and raw mutation rates for transient misalignment hotspots 12

Figure S7. Mutations that occurred repeatedly across independent lineages or experiments 13

Figure S8. The AC_{3+} hotspot is influenced by the 5' nucleotide 14

Figure S9. Strand-dependent sequence context nucleotide frequencies 15

Figure S10. Strand bias of GC_{3+} and AC_{3+} hotspots 16

Figure S11. The G:C→C:G bias towards GC-rich regions is not exclusive to GC_{3+} mutations 17

Figure S12. Distribution of GC-content values for the ±1 to ±100 bp contexts 18

Figure S13. The GC_{3+} hotspot is not seen in strains that constitutively express the SOS response 19

SUPPLEMENTARY TABLES **20**

Table S1. Mutation accumulation experiments used in this study 20

Table S2. Variation between experiments within the same repair group 21

Table S3. Linear models comparing leading versus lagging strand context effects 22

SUPPLEMENTARY REFERENCES **23**

SUPPLEMENTARY METHODS

Collating mutation accumulation data

We compiled base pair substitution (BPS) data from six studies (1–6) that performed mutation accumulation (MA) with *Escherichia coli*. The wild-type (WT) strain was PFM2, a prototrophic derivative of *E. coli* K-12 MG1655 (NC_000913.3) that carries a functional copy of *rpoS*, as described in Lee *et al*. 2012 (1). Most of the experiments were completed by Patricia Foster's lab, and all used the same procedure and media conditions. Briefly, single colonies were isolated on Luria-Bertani agar plates and used as founders for distinct MA lineages (1). Every day, each lineage was re-streaked on an LB agar plate and incubated for 24 hours at 37 °C, then a single colony was randomly selected to continue the lineage. After many generations, a final colony from each lineage was sequenced to identify all the mutations that had arisen. The number of generations per passage was estimated from the diameter of the colonies. In mutation accumulation studies, using rich media and passaging only a single colony (making the effective population size equal to one) is done to minimize the influence of selection on which mutations become fixed. The ratio of synonymous to non-synonymous mutations in these studies does suggest that selection was minimal (1, 3). However, it is worth noting that selection acting during colony growth can still introduce a degree of bias into mutation accumulation experiments (7).

Grouping strains according to proofreading and MMR proficiency

While all strains used in this study were derived from *E. coli* PFM2, most carried deletions of one or more DNA repair genes. We combined data from different strains based solely on their capacity for DNA proofreading and mismatch repair **(Table 1, Table S1)**, which are the main pathways responsible for preventing BPS (8). This grouping strategy is consistent with previous analyses of trinucleotide sequence context (5, 9), and mutational spectra were highly consistent between strains within the same repair group **(Fig. S1)**. Furthermore chi-squared tests on BPS counts across independent experiments show that the variation between different strains within the same repair group was comparable to the variation between different experiments performed with the same strain **(Table S2)**, supporting our decision to aggregate data based on proofreading and MMR proficiency. However, grouping these strains may have obscured some sequence context effects that are dependent on repair pathways other than proofreading or MMR.

The proofreading(+) MMR(+) group included WT *E. coli*, and variants lacking *uvrA*, *nfi*, *ada*, *ogt*, *alkA*, *tagA*, *umuDC*, *dinB*, or *polB*. These genes are involved in nucleotide excision repair (*uvrA*), transcription coupled repair (*uvrA*), repair of deaminated bases (*nfi*), repair of alkylated bases (*ada*, *ogt*, *alkA*, *tagA*), error-prone DNA synthesis (*umuDC*, *dinB*), and DNA synthesis during UV-damage repair (*polB*). Because these genes are generally only active in response to stress or exogenous DNA damage, knocking them out does not significantly influence the mutational spectrum or overall BPS rate in MA experiments (3) **(Fig. S1, Table S2)**.

The proofreading(+) MMR(-) strains all lack at least one of the three genes required for mismatch repair: *mutS*, *mutL*, and *mutH*. Some of the strains also have error prone

polymerases (*umuDC* and *dinB*) or the transcription-repair coupling factor (*mfd*) knocked out. Loss of these genes has a minimal influence on the mutation rate or spectrum in an MMR-deficient background (5) **(Fig. S1, Table S2)**.

The proofreading(-) data came from strains carrying the mutD5 mutation (T15I) in *dnaQ*, which encodes the proofreading subunit of DNA polymerase III. This mutation reduces the polymerase's proofreading capacity by 98%, but otherwise maintains its structure and function (10). Two of the proofreading(-) MMR(+) strains also lack *dinB* or *dinB* and *umuDC*, which has a minimal impact their mutational rate or spectrum (4) **(Fig. S1, Table S2)**. The proofreading(-) MMR(-) data comes from only one strain, which carried the mutD5 mutation of *dnaQ* and a knockout of *mutL*, preventing both proofreading and MMR activity.

We calculated BPS rates using the same method as the original MA studies (1). We pooled mutation and generation counts for each repair background and used this equation: $\frac{\text{number of mutations}}{\text{number of generations} \times \text{base pairs in genome (4,639,675)}}$. To statistically compare mutational spectra between repair groups, we performed a chi-squared test on the contingency table of mutation counts for each BPS type. To show that the A:T versus G:C transition bias did not differ significantly between proofreading(+) MMR(+) and proofreading(-) MMR(-) strains, we conducted a two-sample chi-squared test for equality of proportions.

The entire collated BPS dataset is available in **Supplementary Data 1**. As in the original papers, the genomic position of each mutation maps to the *Escherichia coli* K-12 MG1655 reference sequence (NC_000913.3).

Evaluating sequence context nucleotide frequencies

Typically, sequence context biases are determined by comparing the BPS rate at each unique motif of a given length. In most studies, the motifs are only 3 bp long, i.e., the focal mutating base and the two immediately adjacent bases. This is because as the motif length increases, the number of unique possibilities grows exponentially, and many motifs would have zero mutations. To overcome this problem, we considered each context position surrounding the focal base independently, rather than as part of a specific motif. We counted the frequency of each nucleotide (A, T, G, or C) at each context position across *all* mutations, which meant we had the statistical power to investigate position-specific context effects at any distance. In **Figure 1**, we chose to only display positions ±6 bp from the focal base because they had the strongest influence on mutational bias. Because we evaluated each context position independently, this analysis did not directly identify motifs with elevated mutation rates. Instead, it revealed broader trends in how the extended sequence context influenced mutagenesis and motivated subsequent analyses of specific mutagenic motifs.

To evaluate the extended sequence context, we first needed to determine which context positions to consider as 5' versus 3' of the mutation site. This choice is arbitrary, because we do not know which strand had the original mispair that caused a mutation at a given base pair. However, for consistency, here we always orient using the purine (A or G) as the focal nucleotide, as opposed to the pyrimidine (T or C). Nucleotides that we write on the

left (also denoted as '-') are 5' of the mutation site's purine, while nucleotides written on the right (or '+') are 3' of the purine. Note that orienting by the pyrimidine would provide the same results, just reverse complemented.

To calculate context nucleotide frequencies, we divided the number of times a nucleotide was present at a given context position by the total number of mutations in the dataset – separately for all six BPSs in the four DNA repair backgrounds **(Fig. 1B)**. To prevent the results from being skewed by the sequence of the genome itself, we weighted the nucleotide frequencies from mutation sites by the overall nucleotide frequencies of the context around every site in the genome **(Fig. S2)**. We also used these genomic nucleotide frequencies in chi-squared tests, to determine if the nucleotide frequencies at a given context position are significantly different ($p < 0.05$) than the frequencies expected from the genome. We used the false discovery rate method to correct for multiple comparisons; **Supplementary Data 2** includes the test statistic, raw and corrected *p* values for every test.

The datasets all have vastly different numbers of mutations, from 77 for G:C→C:G mutations in proofreading(-) MMR(-) to 27,770 for A:T→G:C mutations in proofreading(+) MMR(-). As such, while the chi-squared *p*-value indicates if a given context position's contribution to mutational bias is significant, it does not reflect the relative strength of that contribution. We therefore quantified how much the observed nucleotide frequencies at each context position deviated from the expected frequencies **(Fig. 1C)**. This was calculated as the sum of the absolute difference between the observed and expected frequency for each nucleotide: $\sum_{n\in\{A,T,G,C\}} \left|f_{obs}(n) - f_{exp}(n)\right|$, where f_{obs} is the observed frequency and f_{exp} is the expected frequency. The theoretical maximum value is 1.5, corresponding to one nucleotide at 100% frequency and the other three at 0% frequency.

Comparing leading versus lagging strand template

DNA polymerase always synthesises the nascent DNA strand in a 5' → 3' direction, which means traveling along the template DNA strand in a 3' → 5' direction. For one strand (the leading strand template, LDST) the polymerase binds once and travels continuously in the same direction as the replication fork. For the other strand (the lagging strand template, LGST) replication is discontinuous because the polymerase must travel in the opposite direction from the proceeding replication fork, frequently dissociating and rebinding as more of the LGST becomes available to replicate. Because any mutation has been fixed in the genome by the time it is sequenced, it is unknown whether the original mispair occurred during replication of the LDST or the LGST.

However, we do know which base (the purine or the pyrimidine) is on the LDST or LGST for a given base pair. To evaluate how strand influences mutagenesis, for every mutation site we determined if the purine was on the LDST or the LGST. Because bacterial genomes are circular and replicated bidirectionally from a consistent point of origin, the chromosome is split into two 'replichores' – the LDST for one replichore serves as the LGST for the other replichore, and vice versa. For the annotated reference sequence of *E. coli* K-12 MG1655, the replication origin is at 3,925,696 bp and the replication terminus is at 1,640,202 bp. While the exact locations of the origin and terminus vary slightly between different rounds

of replication, we used these values to split the genome into the left and right replichores. To calculate strand-dependent mutation rates, we divided the number of mutations at sites with a purine on the LDST by the number of those sites across the genome, then did the same for sites with a purine on the LGST.

To evaluate how having the purine on the LDST versus LGST influences sequence context effects, we compared the weighted nucleotide frequencies of the ±6 bp flanking positions **(Fig. 4B-D)**. We used linear models to determine the line of best fit for each plot in **Fig. 4C** and calculate their slope and R^2 values **(Table S3)**.

Calculating mutation rates at mononucleotide runs

The nucleotide frequency analysis indicated that specific mononucleotide runs were hotspots for certain BPS mutations. To contextualize the number of mutations appearing at a given mononucleotide run with how prevalent that run is in the genome, we calculated site-specific mutation rates for different classes of mononucleotide run as: $\frac{\text{number of mutations at sites with a given run class}}{\text{number of generations} \times \text{genomic count of sites with that run class}}$. For the $\underline{G}C_{3+}$ and $\underline{A}C_{3+}$ hotspots associated with a 3' run of Cs **(Fig. 3, Fig. S8)**, the mutation sites were classified by the run length of consecutive 3' Cs and the identity of the 5' nucleotide. For **Figure S10**, the run sites were instead classified by run length and whether the purine was on the LDST or LGST.

To identify potential hotspots at consecutive runs of *any* nucleotide, we classified run sites by their length and whether the site was consistent with transient misalignment of the template or nascent strand **(Fig. 2)**. Both events are theoretically possible at any given run: transient misalignment of the template strand causes a BPS mutation at the terminal base within the run, whereas misalignment of the nascent strand produces a mutation at the base immediately downstream of the run **(Box 1)**. In either case, which BPS occurs depends on the identity of the run nucleotide and the immediately adjacent nucleotide. We therefore looked at the position of each mutation and determined if the BPS that occurred is consistent with transient misalignment of the template or nascent strand. A mutation site can be inconsistent with transient misalignment for four reasons: (i) The site is not within or adjacent to a run of ≥2 bp; (ii) the site is within a run but is not the terminal base; (iii) the site is adjacent to a run but is not downstream of the run; (iv) the site *could* be consistent with transient misalignment, but the BPS that occurred is not what transient misalignment would have caused.

However, which site is the terminal base in a run, and which adjacent site is downstream of that run, depends on which strand is being replicated. Because whole genome sequencing does not tell us which strand the mispair happened on, we needed to repeat the analysis twice. First, we categorized every mutation site assuming the strand with a purine always templated the mispair **(Fig. 2B)**, then we categorized the same mutation sites now assuming the pyrimidine always templated the mispair **(Fig. 2C)**. If a specific run motif (e.g. $\underline{G}C_{3+}$) is a hotspot for mutations that are consistent with transient misalignment, it indicates which strand the mispair most likely happened on, because the run is only upstream on one strand.

Determining how regional GC-content influences mutagenesis

To explore sequence context effects beyond the ±6 bp either side of mutation sites, we quantified GC content (GC%) rather than the frequency of specific nucleotides. This reduced the complexity of the data to allow identifying trends at greater distances. We examined progressively greater distances from the mutation sites by calculating the average GC% of a 20 bp sliding context window **(Fig. 5A)**. We then compared these values to the overall GC% of the genome. To determine the genomic regions with an excess of mutations, we took the distribution of GC% values from the -1 to -100 and +1 to +100 context regions and compared them to the distribution of GC% values for same regions around every A and G in the genome **(Fig. 5B, Fig. S12)**. We performed Kolmogorov–Smirnov tests with False Discovery Rate correction to statistically compare the two distributions to this null expectation.

Programs and packages

All analyses were conducted in R (*v4.3.*2) (11) using RStudio (12) with the packages: *tidyverse* (13), *magrittr* (14), *seqinr* (15), *cowplot* (16), *ggtext* (17), and *scales* (18).

SUPPLEMENTARY FIGURES

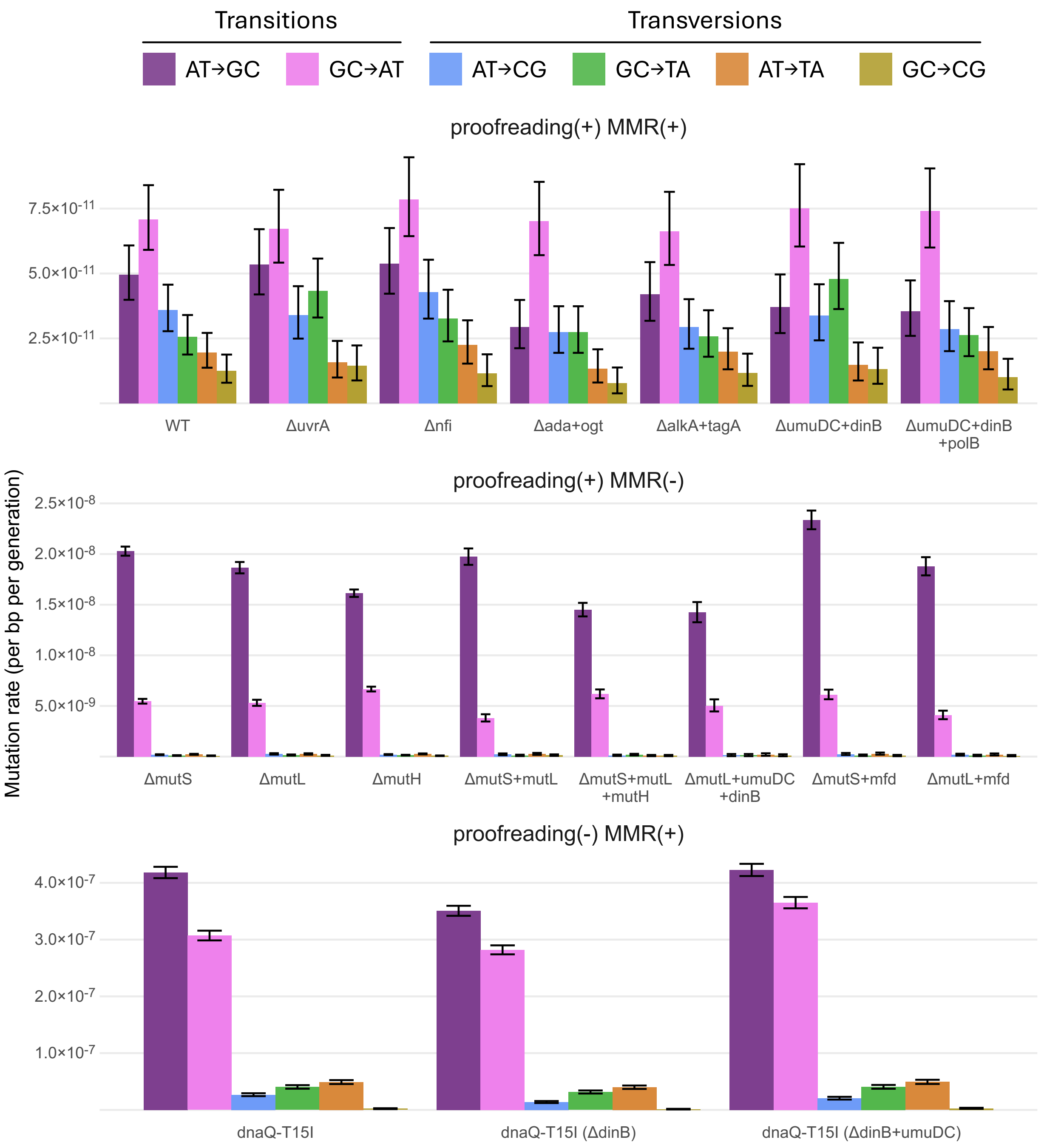

Figure S1. Mutational spectrum of the strains comprising each DNA repair group

We combined data from five published studies (1–6) that performed MA with *E. coli* PFM2 (WT) carrying deletions of different DNA repair genes **(Table S1)**. This figure shows the BPS rates (per base pair per generation) for different strains that were grouped together into the same repair background. Uncertainty (shown as error bars) was calculated using exact 95% Poisson confidence intervals on the pooled mutation counts. The MA data from these strains have been grouped in previous analyses of mutational spectra and trinucleotide sequence context (5, 9). Furthermore, we performed chi-squared tests to determine if the mutational spectra varied significantly between strains in each repair group **(Table S2)**. The tests indicate that variation between different deletion strains from the same repair group was comparable to the variation between independent experiments performed with the same strain. The proofreading(-) MMR(-) group is not shown above because only one strain is included: *dnaQ*-T15I (Δ*mutL*) – **Table 1** shows the mutational spectrum for this strain.

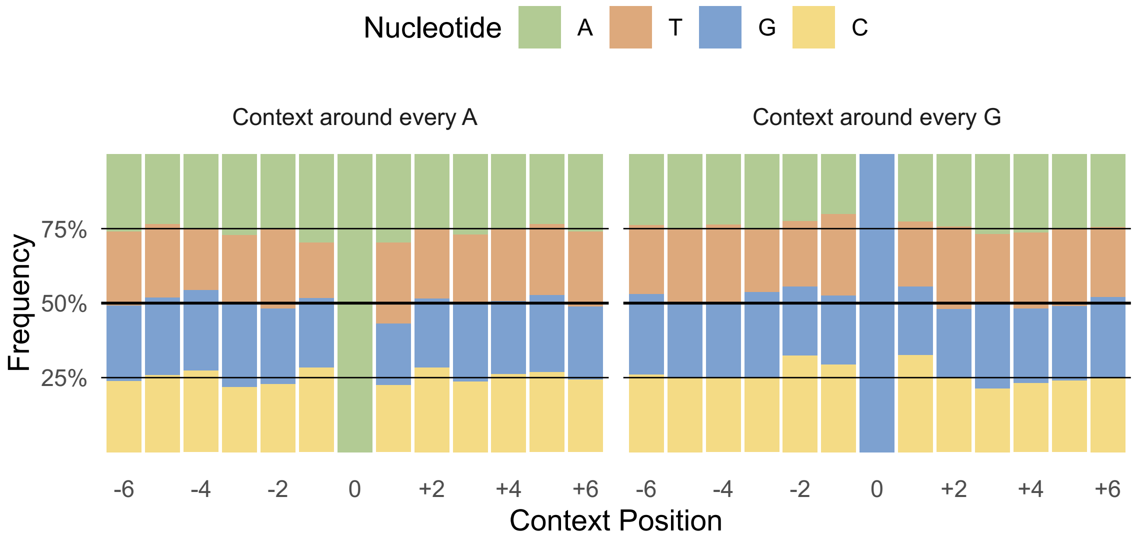

Figure S2. Average genomic sequence context
The frequency of each nucleotide in the ±6 bp sequence context across every site in the genome. Because we always orient relative to the purine, A:T sites have A as the focal nucleotide and G:C sites have G as the focal nucleotide (position '0'). We used these position-specific genomic nucleotide frequencies as the expected values in chi-squared tests for the nucleotide frequencies of the sequence context around mutation sites (see *Methods: Evaluating sequence context nucleotide frequencies*). We also used the genomic nucleotide frequencies to weight the mutation site nucleotide frequencies in **Fig. 1B**, so that the 'null' frequency for each nucleotide was 0.25.

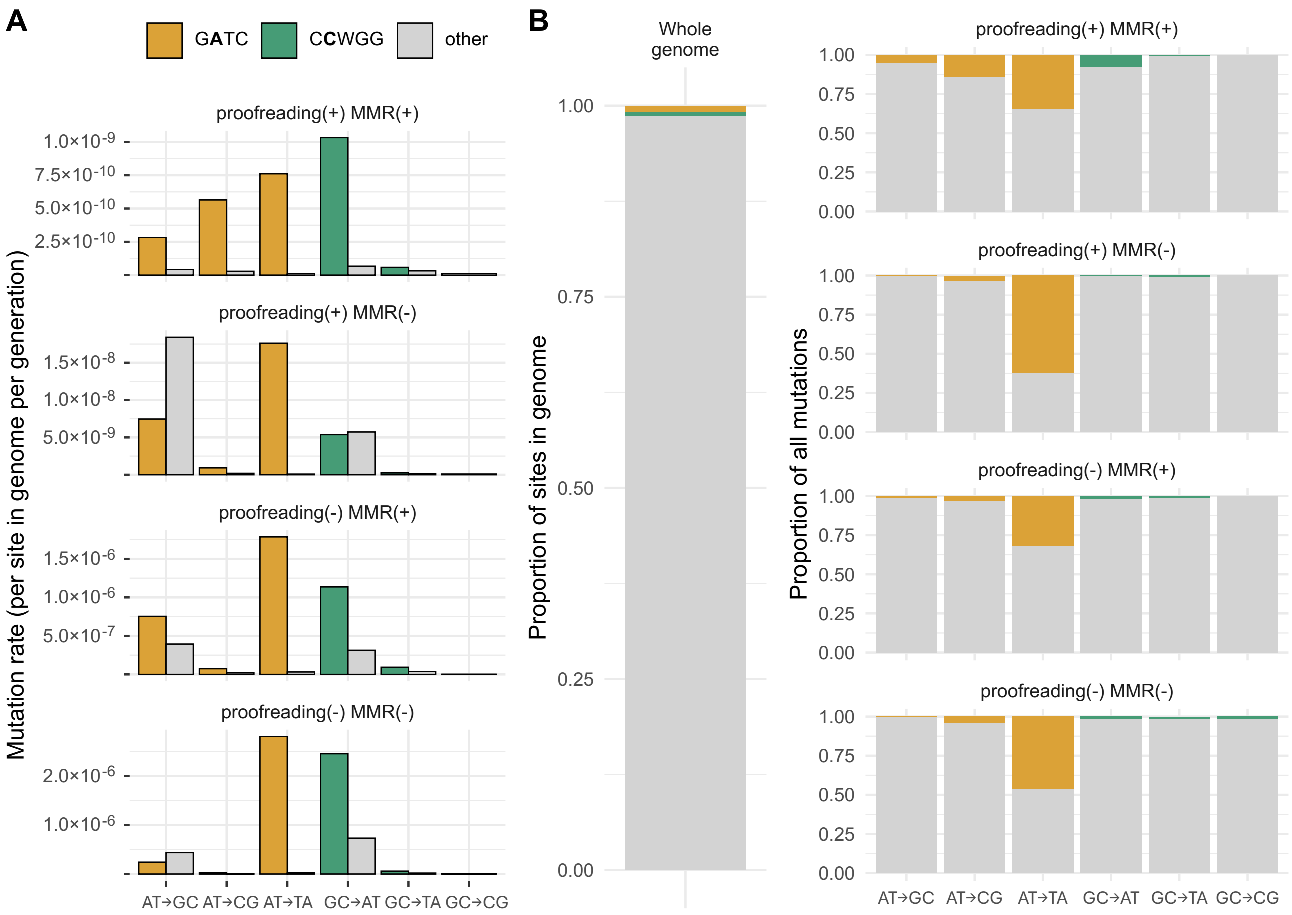

Figure S3. Rate and proportion of mutations at GATC and CCWGG hotspots

The mutation rate **(A)** and proportion of mutations **(B)** at the sequence motifs that are targeted by Dam (orange) and Dcm (green) methylases. The 'whole genome' bar indicates what percentage of base pairs in the genome are the focal site in these mutational hotspots.

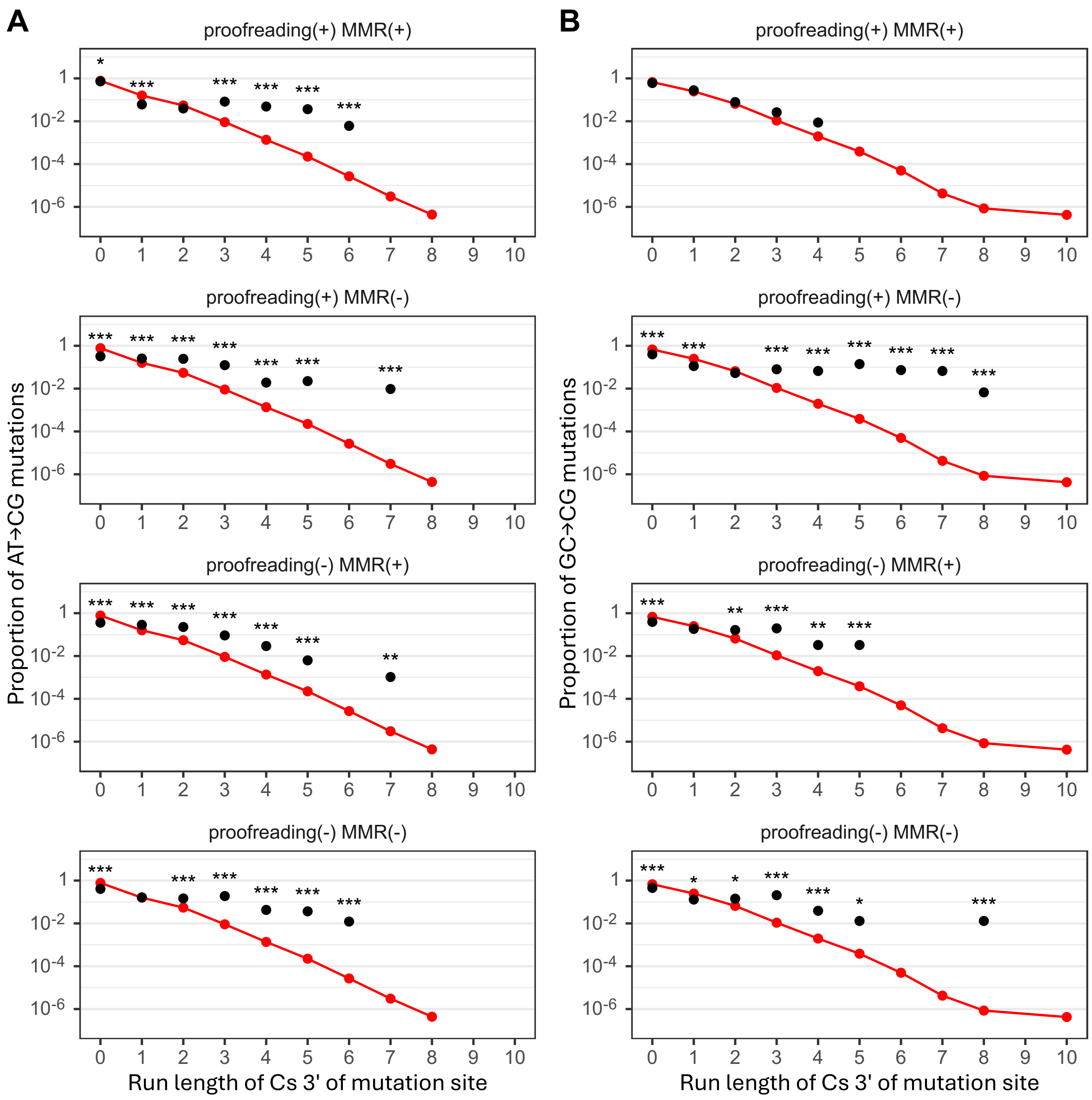

Figure S4. Proportion of mutations at <u>A</u>C$_n$ and <u>G</u>C$_n$ sites

Black dots show the proportion of **A)** A:T→C:G mutations or **B)** G:C→C:G mutations at sites with different numbers of consecutive C nucleotides 3' of the mutated purine. The red dots show the proportion of all A or G nucleotides in the genome with x-number of consecutive 3' Cs. Stars indicate if the proportion of observed mutations is significantly different from the proportion expected by the genome (***: $p < 0.001$, **: $p < 0.01$, *: $p < 0.05$). We used exact binomial tests followed by FDR correction for multiple comparisons.

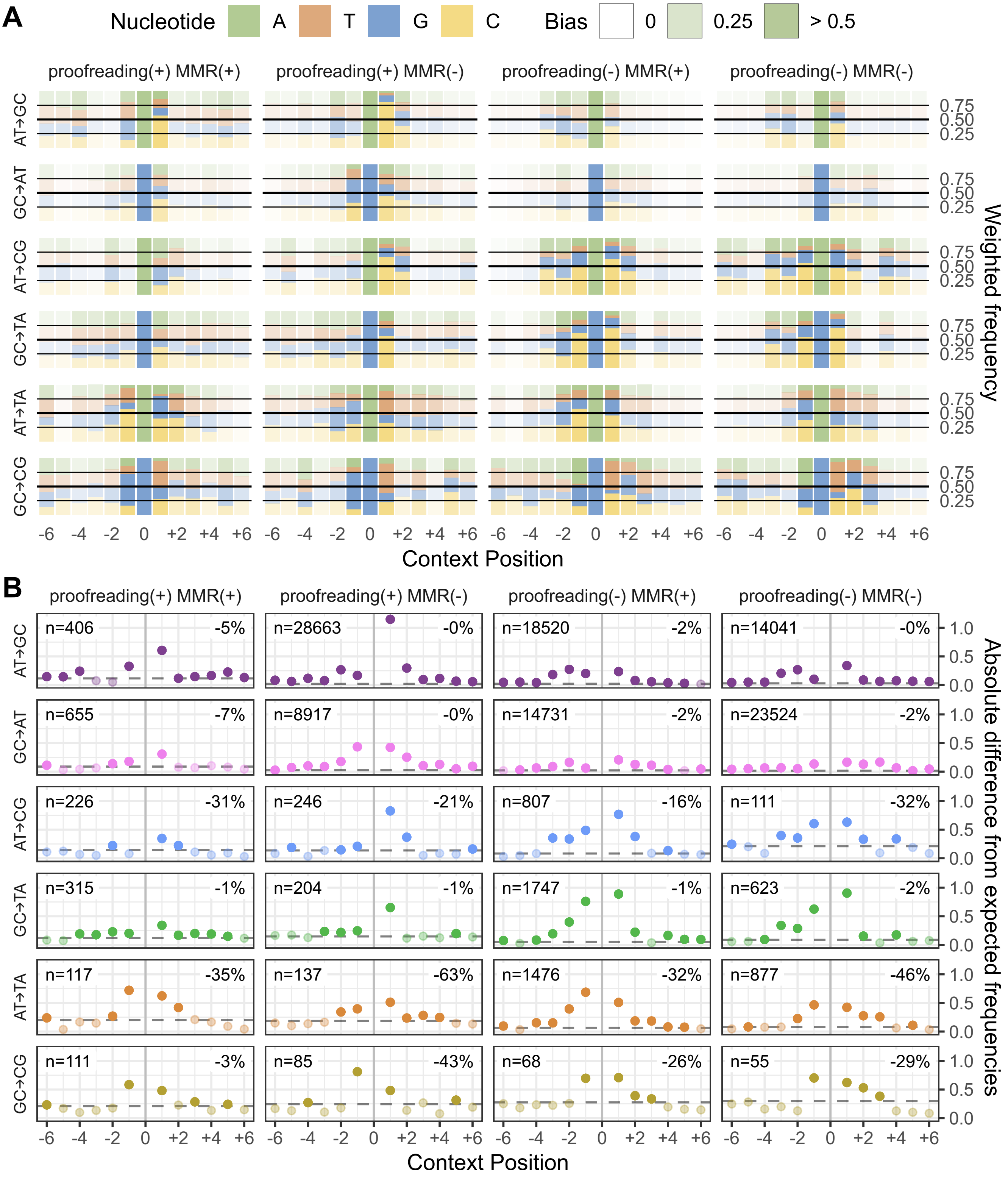

Figure S5. Nucleotide frequencies after excluding the strongest mutational hotspots

We recreated **Fig. 1** after removing all mutations associated with the identified hotspots: the GATC and CCWGG methylation sites, and the AC$_{3+}$ and GC$_{3+}$ mononucleotide run sites. **A)** Nucleotide frequencies at each context position around mutation sites, weighted by the expected nucleotide frequencies across the genome. **B)** The level of bias at each context position, calculated as the sum of absolute differences between observed and expected frequencies. The number of mutations in each dataset is shown, and the percentage value reflects how many hotspot mutations were removed from the original dataset in **Fig. 1**. For some BPS types / repair backgrounds, no mutations occurred at one of these hotspots, making the value 0%.

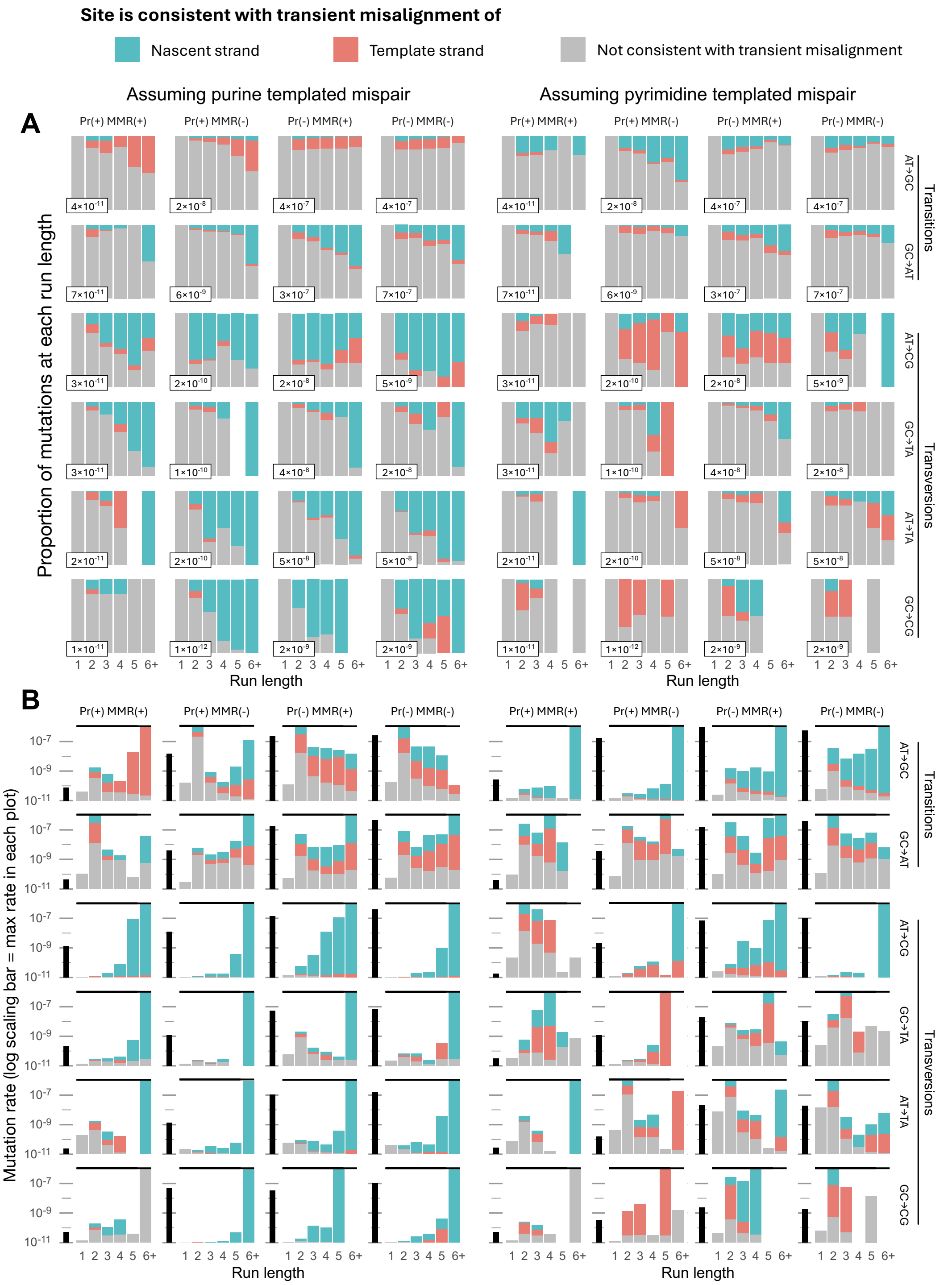

Figure S6. Proportions and raw mutation rates for transient misalignment hotspots

As in **Figure 2**, every mutation site was categorized by whether it is consistent with transient misalignment. Each site was categorized twice, one assuming a purine (A or G) templated the mispair, once assuming a pyrimidine (T or C) templated the mispair. **A)** The proportion of mutations in each category at different run lengths. The number is the bottom left corner of each plot is the overall mutation rate across the entire genome for that BPS in that repair group. **B).** The per-site mutation rate for each of the categories at different run lengths. Because mutation rates vary dramatically between BPS types and repair groups, the plots do not have a shared axis. Instead, the y-axis for each subplot is a linear scale that extends from 0 up to the highest mutation rate shown in that subplot. The black scaling bar on the left of each subplot, which is on a log scale, indicates the actual value of the highest mutation rate.

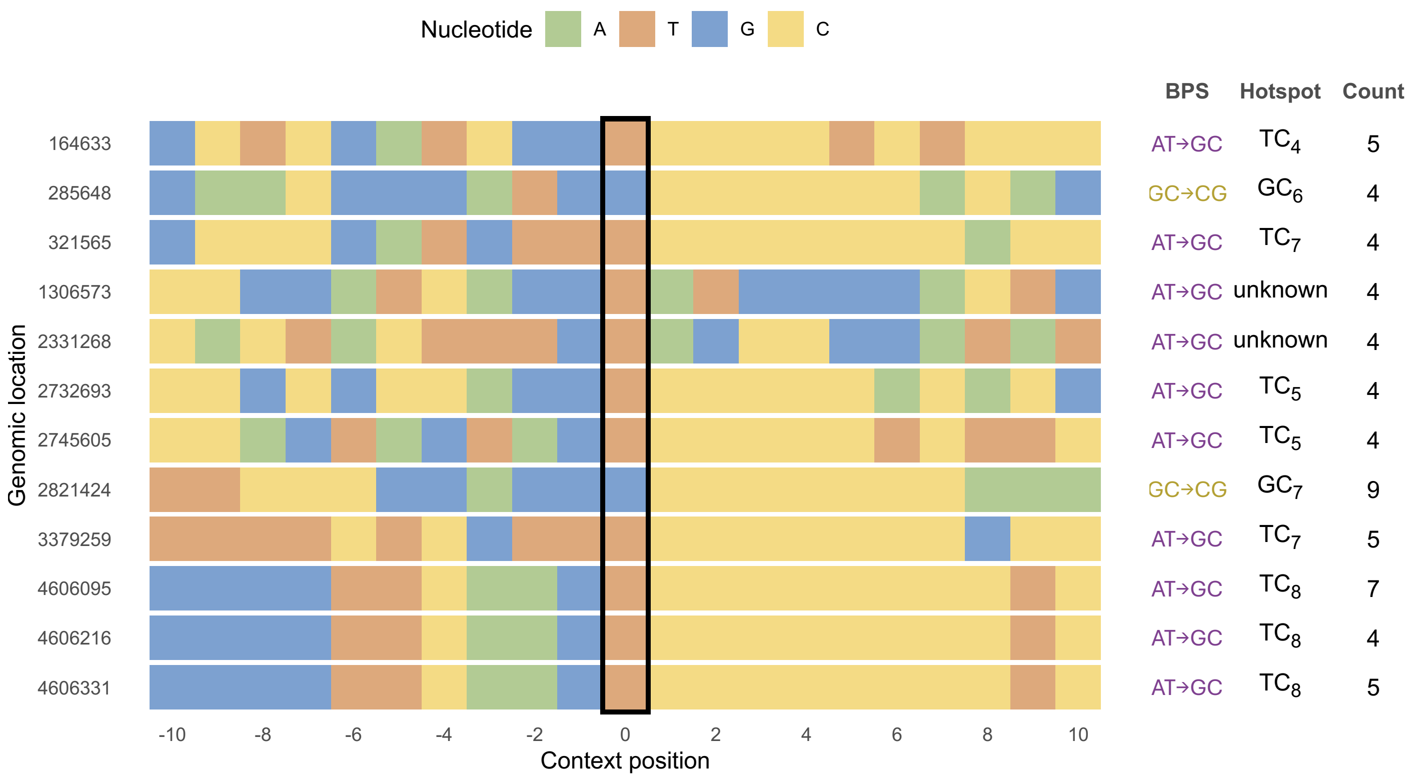

Figure S7. Mutations that occurred repeatedly across independent lineages or experiments
The sequence context around the 12 sites in the genome where the same substitution arose four or more times across independent MA lineages – including all strains regardless of DNA repair proficiency. Most of these hotspots (10/12) are consistent with transient misalignment of the nascent strand, either A:T→G:C transitions at T̲C$_{3+}$ sites, or G:C→C:G transversions at G̲C$_{3+}$ sites. In one case, a single intergenic genomic locus (4,606,095 to 4,606,331 bp) contains three repeated T̲C$_{8}$ sites, that were all mutated multiple times.

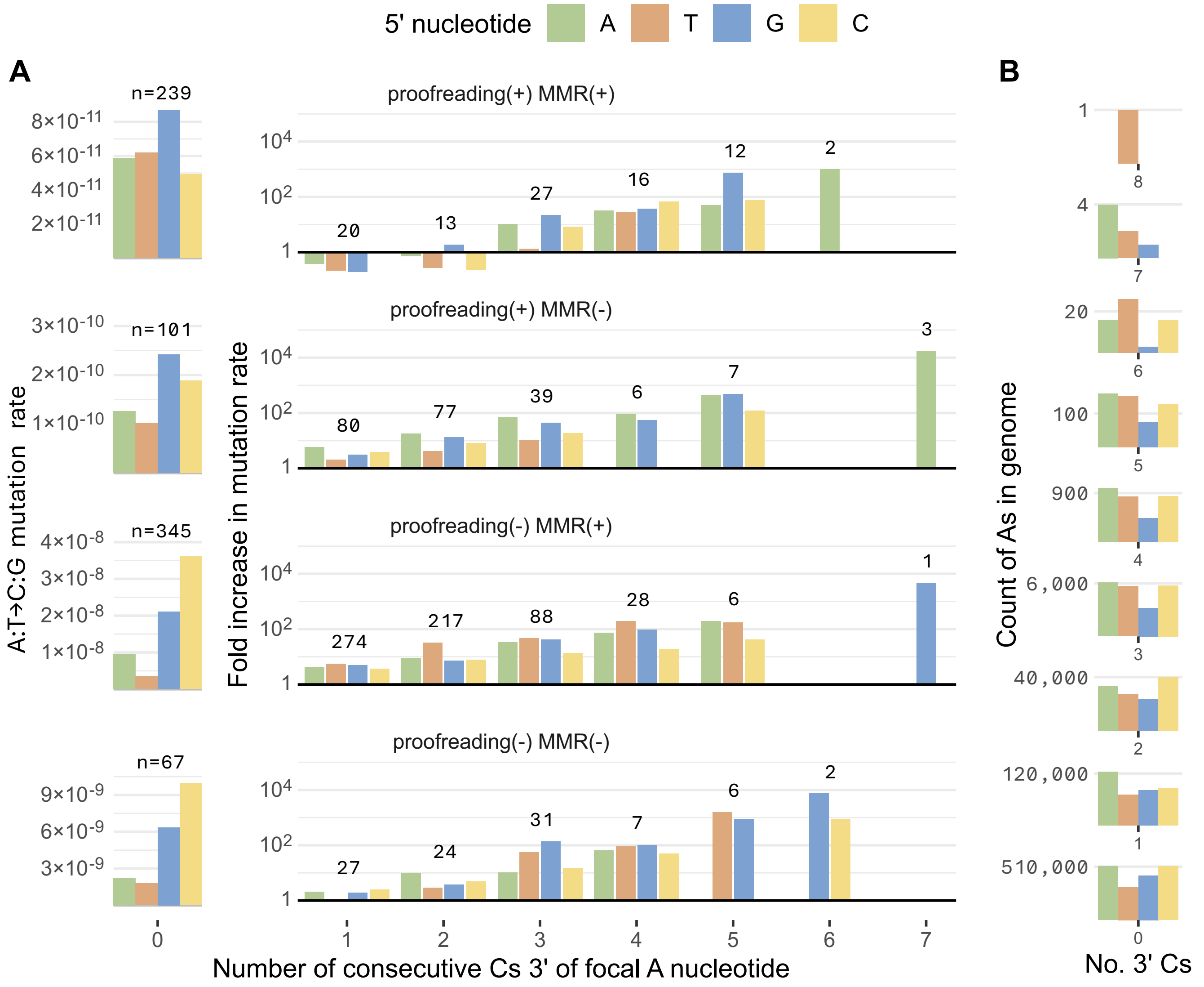

Figure S8. The $\underline{A}C_{3+}$ hotspot is influenced by the 5' nucleotide

Identical to **Fig. 3**, but for A:T→C:G transversions instead of G:C→C:G transversions. **A)** The left-most plots show the A:T→C:G mutation rate (per site per generation) at $N\underline{A}C_0$ sites, i.e., As without an immediately 3' C. We calculated the rate separately depending on the identity of the 5' nucleotide (color of the bar). The adjacent plots show the fold-increase in mutation rate at $N\underline{A}C_{1+}$ sites, relative to the corresponding $N\underline{A}C_0$ site. The x-axis shows the number of consecutive C nucleotides 3' of the focal $\underline{A}$. The number above the bars indicates the total number of A:T→C:G mutations at $\underline{A}C_x$ sites. **B)** The counts of all As in the genome split by the number of consecutive 3' Cs (x-axis) and the identity of the 5' nucleotide (color).

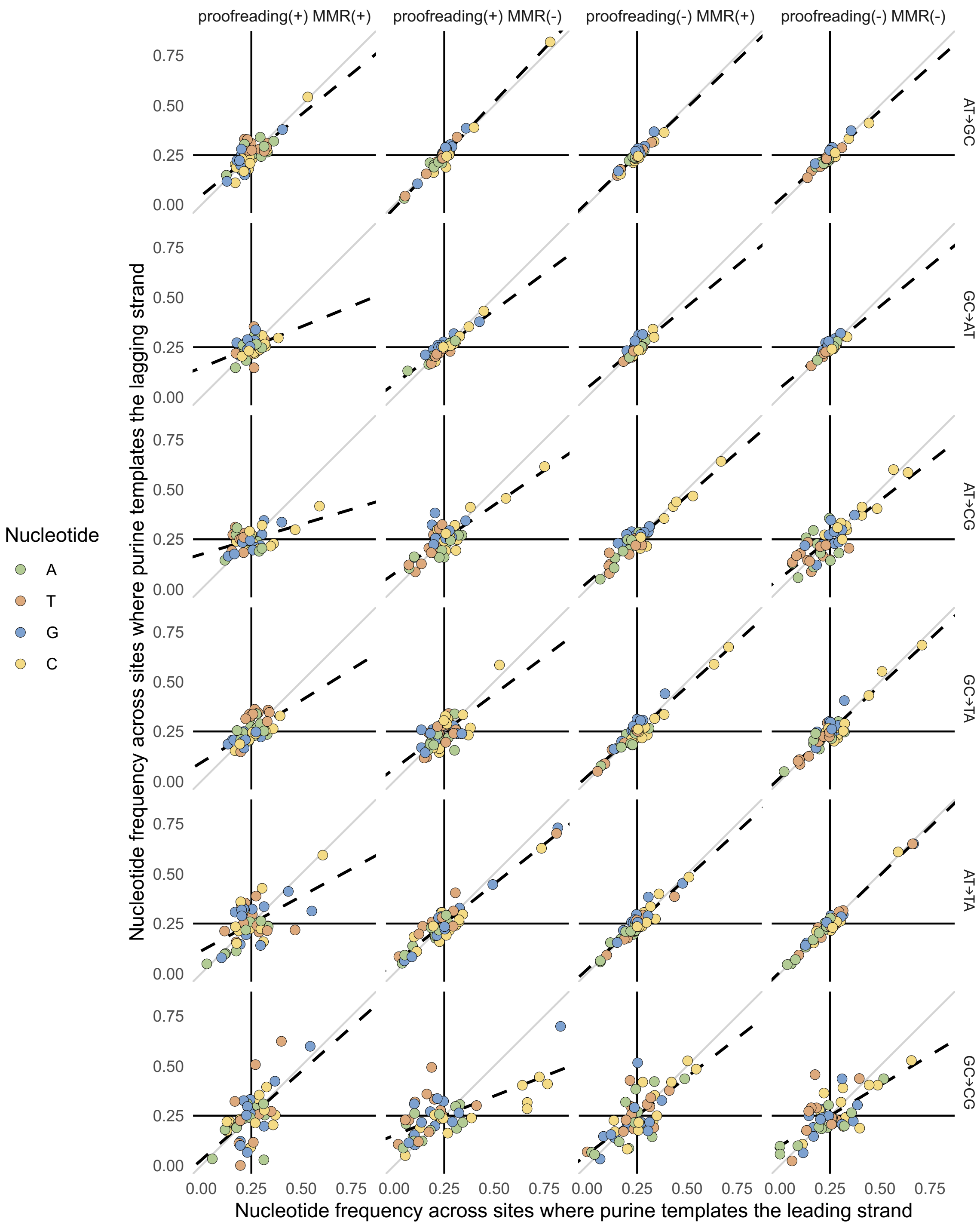

Figure S9. Strand-dependent sequence context nucleotide frequencies

Identical to **Fig. 4C** but showing the data for all BPSs. Each point represents the weighted frequency of one nucleotide (A, T, G, or C) at one position (-6 to +6) across all mutation sites where the purine templates the leading versus the lagging strand. The dashed line represents the line of best fit from linear regression. If the nucleotide frequencies were identical between sites where the purine templates the leading versus lagging strand, then the line of best fit would have a slope of 1 and an R^2 value of 1. Slope and R^2 values for all plots are given in **Fig. 4D** and **Table S3**.

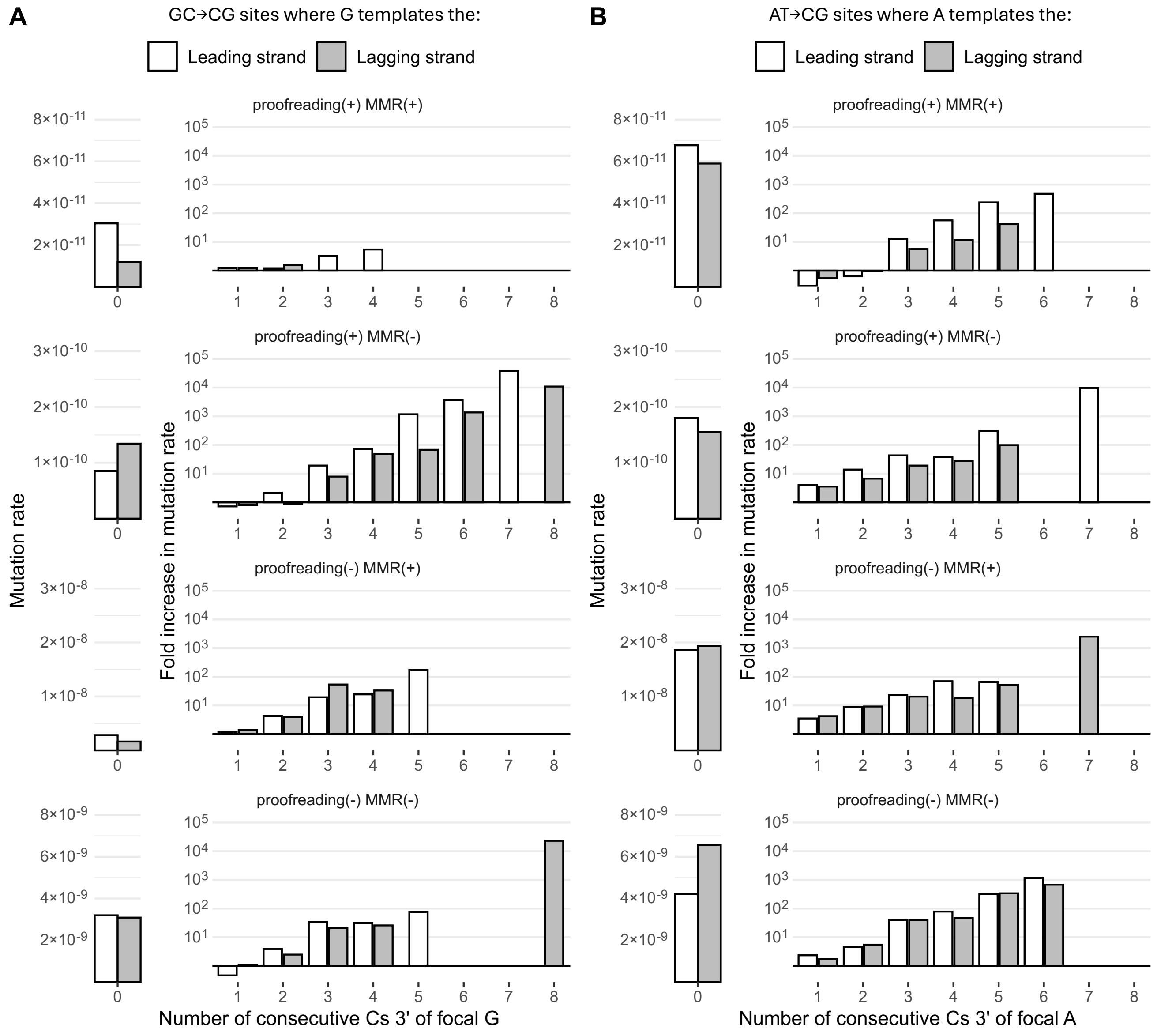

Figure S10. Strand bias of $\underline{G}C_{3+}$ and $\underline{A}C_{3+}$ hotspots

The same plot as **Fig. 3A** and **Supplementary Fig. 6**, except the sites are classified by whether the purine templates leading or lagging strand synthesis, rather than the 5' nucleotide. **(A)** G:C→C:G and **(B)** A:T→C:G mutation rates. The left-most plots give the raw mutation rate (per site per generation) for sites with zero 3' Cs. The plots on the right show the fold increase in mutation rate at sites with one or more 3' Cs, relative to the raw mutation rate for sites with zero 3' Cs. The $\underline{G}C_{3+}$ and $\underline{A}C_{3+}$ hotspots are both consistent with transient misalignment, so we can assume that the purine (G or A) most likely templated the mispair, because the mononucleotide run is upstream (3' on template strand) of the purine **(Fig. 2)**. Therefore, we can also deduce whether transient misalignment and the resulting mispair occurred during leading or lagging strand synthesis. These plots suggest mutations are more likely to arise from transient misalignment when it occurs during leading strand synthesis.

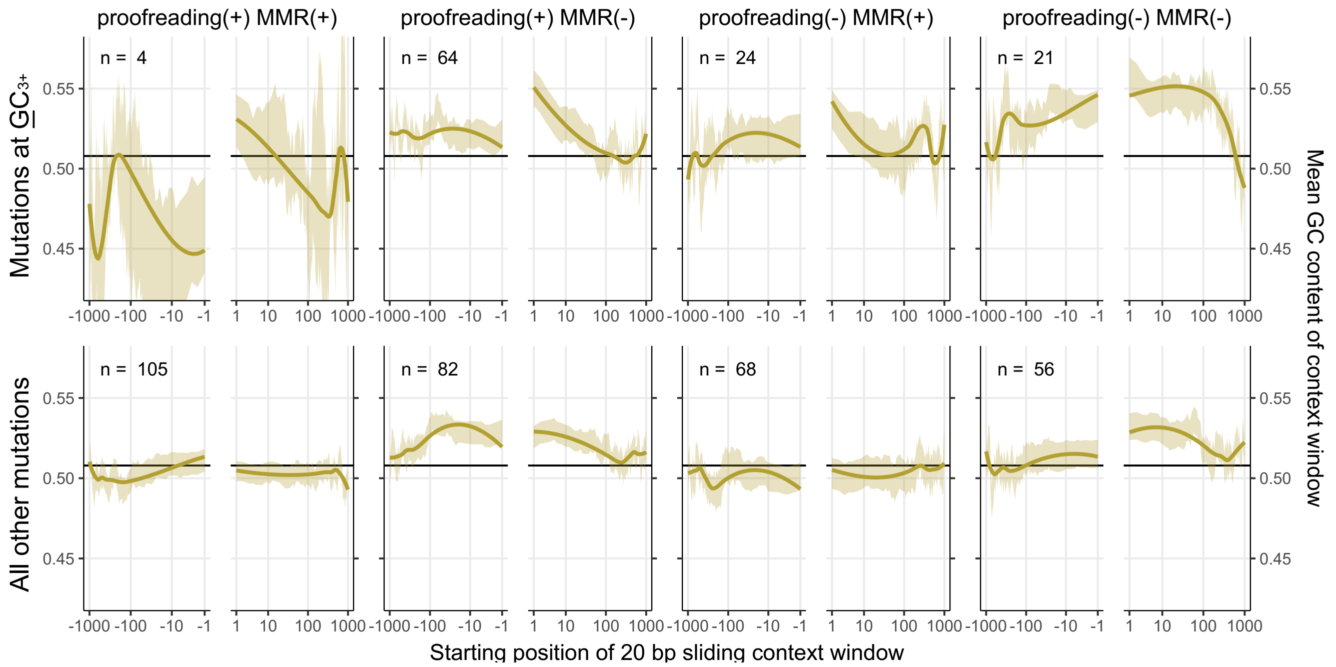

Figure S11. The G:C→C:G bias towards GC-rich regions is not exclusive to $\underline{G}C_{3+}$ mutations

When MMR is absent, G:C→C:G mutations are more common in regions with higher GC% **(Fig. 5A)**. To demonstrate that this is a fully distinct phenomenon from the $\underline{G}C_{3+}$ hotspot for G:C→C:G, we recreated **Fig. 5A** but split between $\underline{G}C_{3+}$ site mutations and all other G:C→C:G mutations. The y-axis shows the mean GC% across all mutation sites of a 20 bp sliding context window that starts from ±1 to ±20 and extends out to ±1000. The shaded ribbon shows the standard error of the mean for each individual context window. The line represents a loess-smoothed curve of the mean GC% values. The horizontal dashed line shows the overall GC% of the of the *E*. coli genome (0.508).

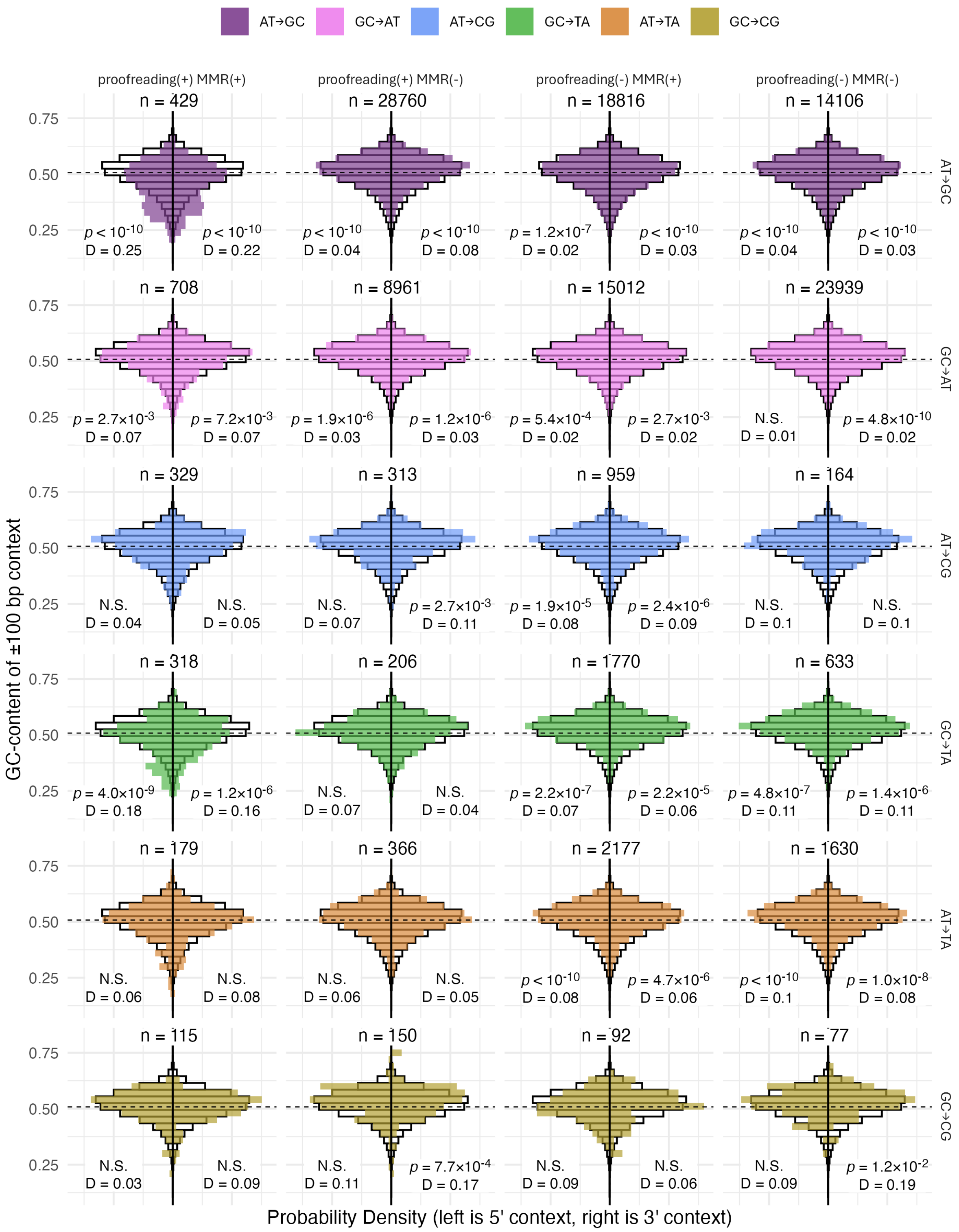

Figure S12. Distribution of GC-content values for the ±1 to ±100 bp contexts

Histograms of the probability density function for GC% values across all mutations for the -1 to -100 (left) and +1 to +100 bp (right) context windows. The white outline represents the null expectation from the average context windows around every site in the genome. We performed Kolmogorov-Smirnov tests with false discovery rate correction to compare the distributions to the null expectation (adjusted *p*-value and test statistic shown).

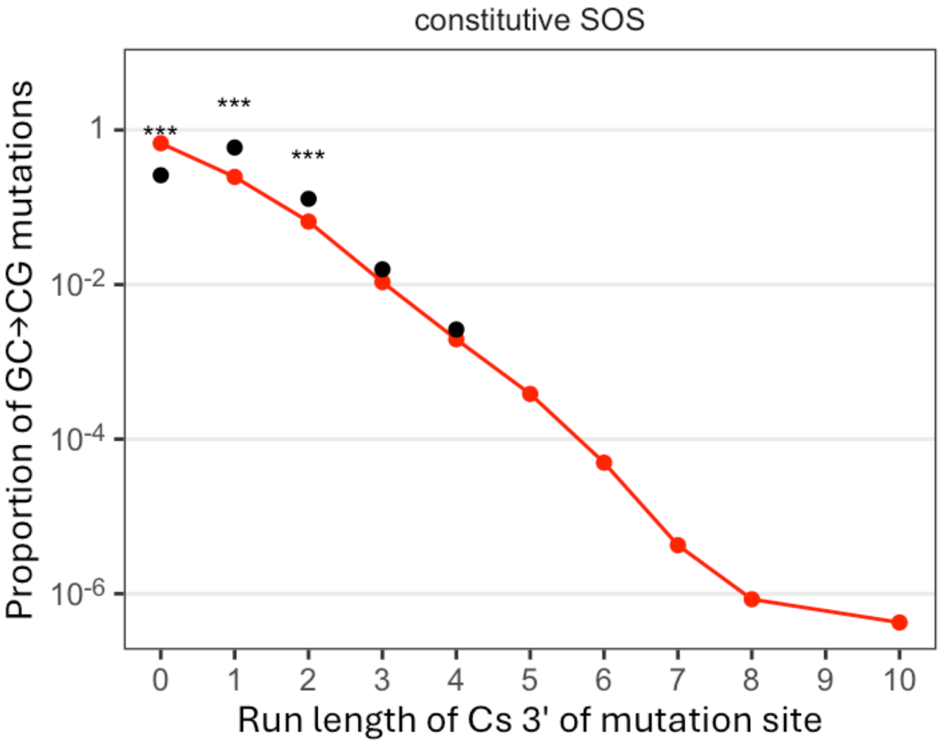

Figure S13. The <u>G</u>C_{3+} hotspot is not seen in strains that constitutively express the SOS response
The same plot as **Fig. S4**, except for strains that constitutively express the SOS response because of an E38K mutation in the *recA730* gene (data from Niccum *et al*. 2020) (19). Shows the proportion of G:C→C:G mutations (black dots) at different 3' C run lengths, compared to the proportion of G sites in the genome with different 3' C run lengths (red dots). Stars indicate if the proportion of observed mutations is significantly different from the proportion expected by the genome. We used exact binomial tests followed by correction for multiple testing using the false discovery rate method.

SUPPLEMENTARY TABLES

group	source	strain	experiment	lines	# of BPS	generations	rate	lower	upper
proofreading(+) MMR(+)	Lee *et al.* 2012	PFM2 (WT)	1	36	93	117040	1.71E-10	1.38E-10	2.10E-10
			2	21	140	133476	2.26E-10	1.90E-10	2.67E-10
	Foster *et al.* 2015	ΔuvrA	1	47	316	298450	2.28E-10	2.04E-10	2.55E-10
		Δnfi	1	47	333	296476	2.42E-10	2.17E-10	2.69E-10
		Δada+ogt	1	48	250	307181	1.75E-10	1.54E-10	1.98E-10
		ΔalkA+tagA	1	47	265	292575	1.95E-10	1.72E-10	2.20E-10
		ΔdinB+umuDC	1	43	269	261354	2.22E-10	1.96E-10	2.50E-10
		ΔdinB+umuDC+polB	1	45	252	279180	1.94E-10	1.71E-10	2.20E-10
	Long *et al.* 2016	PFM2 (WT)	3	35	79	77372	2.20E-10	1.74E-10	2.74E-10
	Tincher *et al.* 2017	PFM2 (WT)	4	27	81	67963	2.57E-10	2.04E-10	3.19E-10
proofreading(+) MMR(-)	Lee *et al.* 2012	ΔmutL	1	34	1625	12750	2.75E-08	2.61E-08	2.88E-08
	Long *et al.* 2016	ΔmutS	1	12	969	9156	2.28E-08	2.14E-08	2.43E-08
	Tincher *et al.* 2017	ΔmutS	2	8	1273	11281	2.43E-08	2.30E-08	2.57E-08
	Foster *et al.* 2018	ΔmutS	3	29	2410	17041	3.05E-08	2.93E-08	3.17E-08
			4	42	2797	21922	2.75E-08	2.65E-08	2.85E-08
			5	41	2763	24148	2.47E-08	2.37E-08	2.56E-08
		ΔmutL	2	25	1350	14367	2.02E-08	1.92E-08	2.14E-08
			3	38	2709	22382	2.61E-08	2.51E-08	2.71E-08
		ΔmutH	1	37	10896	99960	2.35E-08	2.30E-08	2.39E-08
		ΔmutSL	1	43	2845	25270	2.43E-08	2.34E-08	2.52E-08
		ΔmutSLH	1	23	1546	14752	2.26E-08	2.15E-08	2.37E-08
			2	21	1119	12367	1.95E-08	1.84E-08	2.07E-08
		ΔmutS+mfd	1	45	3218	22942	3.02E-08	2.92E-08	3.13E-08
		ΔmutL+mfd	1	32	2126	19554	2.34E-08	2.24E-08	2.44E-08
		ΔmutL+dinB+umuDC	1	23	1110	12078	1.98E-08	1.87E-08	2.10E-08
proofreading(-) MMR(+)	Niccum *et al.* 2018	dnaQ-T15I	1	26	13625	3481	8.43E-07	8.29E-07	8.58E-07
		dnaQ-T15I (ΔdinB)	1	34	12505	3748	7.19E-07	7.06E-07	7.32E-07
		dnaQ-T15I (ΔdinB+umuDC)	1	19	8747	2126	8.86E-07	8.68E-07	9.05E-07
			2	11	3949	911	9.34E-07	9.05E-07	9.63E-07
proofreading(-) MMR(-)	Niccum *et al.* 2018	dnaQ-T15I (ΔmutL)	1	36	26406	4488	1.27E-06	1.25E-06	1.28E-06
			2	21	7326	1309	1.21E-06	1.18E-06	1.23E-06
			3	18	6817	1215	1.21E-06	1.18E-06	1.24E-06

Table S1. Mutation accumulation experiments used in this study

We collated MA data from five published studies: Lee *et al*. 2012 (1), Foster *et al*. 2015 (3), Long *et al*. 2016 (2), Tincher *et al.* 2017 (6), Foster *et al*. 2018 (5), and Niccum *et al.* 2018 (4). We combined data from strains carrying deletions of different DNA repair genes based on the strains' proficiency for proofreading and mismatch repair. For some strains, more than one independent experiment was included, either from the same study or different studies. For each experiment, the table shows: total number of BPS mutations, number of replicate lineages that were sequenced ('lines'), total number of generations across all lineages, the overall BPS rate (mutation count per base pair per generation) and the 95% Poisson confidence intervals. The variation between independent experiments from the same strain or in the same repair group is shown in **Table S2**.

	repair group	strain(s)	number of experiments	n (total mutations)	χ^2	degrees of freedom	*p* value	k	Cramér's V
across one group	proofreading(+) MMR(+)	all in group	10	2,078	57.1	45	0.11	6	0.07
	proofreading(-) MMR(+)	all in group	15	38,756	570.8	70	1.3E-79	6	0.05
	proofreading(+) MMR(-)	all in group	4	38,826	182.0	15	9.3E-31	4	0.04
	proofreading(-) MMR(-)	group only includes dnaQ-T15I (ΔmutL)	3	40,549	1073.5	10	2.77E-224	3	0.12
across one strain	proofreading(+) MMR(+)	WT	4	393	21.0	15	0.14	4	0.13
	proofreading(+) MMR(-)	ΔmutS	5	10,212	32.2	20	0.04	5	0.03
		ΔmutL	3	5,684	63.6	10	7.5E-10	3	0.07
		ΔmutSLH	2	2,665	16.4	5	0.01	2	0.08
	proofreading(-) MMR(+)	dnaQ-T15I (ΔdinB+umuDC)	2	12,696	89.4	5	8.9E-18	2	0.08
	proofreading(-) MMR(-)	dnaQ-T15I (ΔmutL)	3	40,549	1073.5	10	2.77E-224	3	0.12

Table S2. Variation between experiments within the same repair group

We performed chi-squared tests to assess differences in mutational spectra between independent experiments within the same DNA repair group. For each group, contingency tables were constructed from counts of each BPS type across every experiment included in that group (upper part of table). We also performed tests to compare independently conducted experiments from the same *E. coli* strain, for the limited number of strains where we had data from more than one experiment (lower part of table). Statistics for the three dnaQ-T15I (Δ*mutL*) experiments (grey shading) are included in both the upper and lower part because this was the only strain included in the proofreading(-) MMR(-) group. Because the total number of mutations (n) varies substantially between different repair groups, direct comparison of χ^2 statistics and associated *p* values is not informative. We therefore quantified effect sizes by calculating Cramér's V, where $V = \sqrt{\frac{\chi^2}{n*(k-1)}}$ and k is the minimum dimension of the contingency table. Cramér's V is between 0 and 1, and larger values indicate greater divergence in mutational spectra between experiments. This metric of effect size shows that variation between strains in the same repair group is not greater than the variation between independent experiments from the same strain.

group	BPS	slope	*p* value (slope)	R^2
proofreading(+) MMR(+)	AT→GC	0.82	9.09E-02	0.61
	GC→AT	0.39	1.19E-04	0.16
	AT→CG	0.30	1.02E-10	0.25
	GC→TA	0.60	8.66E-03	0.33
	AT→TA	0.56	8.43E-04	0.35
	GC→CG	0.86	4.62E-01	0.33
proofreading(+) MMR(-)	AT→GC	1.07	4.03E-02	0.97
	GC→AT	0.73	2.86E-06	0.85
	AT→CG	0.70	5.55E-04	0.67
	GC→TA	0.78	9.75E-02	0.47
	AT→TA	0.79	1.08E-05	0.91
	GC→CG	0.40	2.45E-11	0.49
proofreading(-) MMR(+)	AT→GC	0.95	3.74E-01	0.89
	GC→AT	0.82	1.23E-01	0.56
	AT→CG	0.87	1.54E-02	0.89
	GC→TA	0.90	2.29E-02	0.93
	AT→TA	0.92	1.39E-01	0.89
	GC→CG	0.78	6.76E-02	0.53
proofreading(-) MMR(-)	AT→GC	0.89	4.33E-02	0.88
	GC→AT	0.82	3.66E-02	0.72
	AT→CG	0.78	9.54E-03	0.72
	GC→TA	0.93	1.78E-01	0.89
	AT→TA	0.97	2.35E-01	0.97
	GC→CG	0.61	2.50E-04	0.50

Table S3. Linear models comparing leading versus lagging strand context effects
Gives the slope, associated *p* value (after FDR correction) compared against a slope of 1, and R^2 values for comparing context nucleotide frequencies between sites where the purine templates the leading strand versus sites where the purine templates the lagging strand **(Fig. 4C, Fig. S9)**.

SUPPLEMENTARY REFERENCES

1. H. Lee, E. Popodi, H. Tang, P. L. Foster, Rate and molecular spectrum of spontaneous mutations in the bacterium Escherichia coli as determined by whole-genome sequencing. *Proc. Natl. Acad. Sci. U. S. A.* **109**, E2774 (2012).
2. H. Long, *et al.*, Antibiotic treatment enhances the genome-wide mutation rate of target cells. *Proc. Natl. Acad. Sci.* **113**, E2498–E2505 (2016).
3. P. L. Foster, H. Lee, E. Popodi, J. P. Townes, H. Tang, Determinants of spontaneous mutation in the bacterium Escherichia coli as revealed by whole-genome sequencing. *Proc. Natl. Acad. Sci. U. S. A.* **112**, E5990-5999 (2015).
4. B. A. Niccum, H. Lee, W. MohammedIsmail, H. Tang, P. L. Foster, The Spectrum of Replication Errors in the Absence of Error Correction Assayed Across the Whole Genome of Escherichia coli. *Genetics* **209**, 1043–1054 (2018).
5. P. L. Foster, *et al.*, Determinants of Base-Pair Substitution Patterns Revealed by Whole-Genome Sequencing of DNA Mismatch Repair Defective Escherichia coli. *Genetics* **209**, 1029–1042 (2018).
6. C. Tincher, H. Long, M. Behringer, N. Walker, M. Lynch, The Glyphosate-Based Herbicide Roundup Does Not Elevate Genome-Wide Mutagenesis of *Escherichia coli*. *G3 GenesGenomesGenetics* **7**, 3331–3335 (2017).
7. A. Mahilkar, N. Raj, S. Kemkar, S. Saini, Selection in a growing colony biases results of mutation accumulation experiments. *Sci. Rep.* **12**, 15470 (2022).
8. R. M. Schaaper, Base selection, proofreading, and mismatch repair during DNA replication in Escherichia coli. *J. Biol. Chem.* **268**, 23762–23765 (1993).
9. S. K. Garushyants, *et al.*, Mutational Signatures in Wild Type Escherichia coli Strains Reveal Predominance of DNA Polymerase Errors. *Genome Biol. Evol.* **16**, evae035 (2024).
10. R. M. Schaaper, M. Radman, The extreme mutator effect of Escherichia coli mutD5 results from saturation of mismatch repair by excessive DNA replication errors. *EMBO J.* **8**, 3511–3516 (1989).
11. R Core Team, R: A Language and Environment for Statistical Computing. (2023). Deposited 2023.
12. Posit team, RStudio: Integrated Development Environment for R. (2025). Deposited 2025.
13. H. Wickham, tidyverse: Easily Install and Load the “Tidyverse.” https://doi.org/10.32614/CRAN.package.tidyverse. Deposited 9 September 2016.
14. S. M. Bache, H. Wickham, magrittr: A Forward-Pipe Operator for R. https://doi.org/10.32614/CRAN.package.magrittr. Deposited 25 February 2014.
15. D. Charif, J. R. Lobry, seqinr: Biological Sequences Retrieval and Analysis. https://doi.org/10.32614/CRAN.package.seqinr. Deposited 22 November 2004.
16. C. O. Wilke, cowplot: Streamlined Plot Theme and Plot Annotations for “ggplot2.” https://doi.org/10.32614/CRAN.package.cowplot. Deposited 3 June 2015.
17. C. O. Wilke, B. M. Wiernik, ggtext: Improved Text Rendering Support for “ggplot2.” https://doi.org/10.32614/CRAN.package.ggtext. Deposited 4 June 2020.
18. H. Wickham, T. L. Pedersen, D. Seidel, scales: Scale Functions for Visualization. https://doi.org/10.32614/CRAN.package.scales. Deposited 22 September 2011.
19. B. A. Niccum, *et al.*, New complexities of SOS-induced “untargeted” mutagenesis in Escherichia coli as revealed by mutation accumulation and whole-genome sequencing. *DNA Repair* **90**, 102852 (2020).
